# Technical challenges in REM sleep microstructure classification: A study of patients with REM sleep behaviour disorder

**DOI:** 10.1111/jsr.14208

**Published:** 2024-04-12

**Authors:** C. William Yao, Giuseppe Fiamingo, Karine Lacourse, Sonia Frenette, Ronald B. Postuma, Jacques Y. Montplaisir, Jean‐Marc Lina, Julie Carrier

**Affiliations:** ^1^ Psychology Department Université de Montréal Montréal Québec Canada; ^2^ Center for Advanced Research in Sleep Medicine, Research center of the CIUSS du Nord‐de‐l'Ile‐de‐Montréal Montréal Montréal Québec Canada; ^3^ Department of Brain and Behavioral Sciences, University of Pavia Pavia Italy; ^4^ Department of Neurology and Neurosurgery McGill Montréal Québec Canada; ^5^ McGill University Health Center Montréal Québec Canada; ^6^ Department Psychiatry Université de Montréal Montréal Québec Canada; ^7^ Department of Electrical Engineering École de Technologie Supérieure Montréal Québec Canada; ^8^ Centre de Recherches Mathématiques Université de Montréal Montréal Québec Canada

**Keywords:** rapid eye movement, rapid eye movement sleep disorder, rapid eye movement sleep microstructures, scoring

## Abstract

While commonly treated as a uniform state in practice, rapid eye movement sleep contains two distinct microstructures—phasic (presence of rapid eye movement) and tonic (no rapid eye movement). This study aims to identify technical challenges during rapid eye movement sleep microstructure visual classification in patients with rapid eye movement sleep behaviour disorder, and to propose solutions to enhance reliability between scorers. Fifty‐seven sleep recordings were randomly allocated into three subsequent batches (*n* = 10, 13 and 34) for scoring. To reduce single‐centre bias, we recruited three raters/scorers, with each trained from a different institution. Two raters independently scored each 30‐s rapid eye movement sleep into 10 × fSEM3‐s phasic/tonic microstructures based on the AASM guidelines. The third rater acted as an “arbitrator” to resolve opposite opinions persisting during the revision between batches. Besides interrater differences in artefact rejection rate, interrater variance frequently occurred due to transitioning between microstructures and moderate‐to‐severe muscular/electrode artefact interference. To enhance interrater agreement, a rapid eye movement scoring schematic graph was developed, incorporating proxy electrode use, filters and cut‐offs for microstructure transitioning. To assess potential effectiveness of the schematic graph proposed, raters were instructed to systematically apply it in scoring for the third batch. Of the 34 recordings, 27 reached a Cohen's kappa score above 0.8 (i.e. almost perfect agreement between raters), significantly improved from the prior batches (*p* = 0.0003, Kruskal–Wallis test). Our study illustrated potential solutions and guidance for challenges that may be encountered during rapid eye movement sleep microstructure classification.

## 1 INTRODUCTION

Rapid eye movement (REM) was first featured, along with its concomitant phenomena, in a 2‐year sleep study by Aserinsky and Kleitman (1953). Compared with slow eye movements (SEM), REM events are typically binocularly symmetrical and short‐lived (Aserinsky & Kleitman, 1955). Besides serving as a key criterion for sleep staging since 1959 (Jouvet et al., 1959), REM detection is crucial for differentiating REM sleep microstructures (Moruzzi, 1963; i.e. phasic – with REM; tonic – without REM).

Most human studies treat REM sleep as a uniform state. This common practice could be attributed to the tedious task associated with REM detection and its accompanying challenges. Unlike electroencephalography (EEG) analysis, artefacts in electrooculography (EOG) remain as brief descriptions with little solutions in practice, although challenges were already noted in 1953 (Aserinsky & Kleitman, 1953). Similarly, descriptions of REM in most guidelines primarily focus on its role as a sleep stage transition determinant leaving further descriptions in early studies. These subsequently affected the performance of (semi‐) automated REM detection algorithms and the level of agreement among raters (Yetton et al., 2016).

In comparison to the standard sleep staging, REM sleep microstructures are shorter and more irregular in duration. In addition, the phasic microstructure occupies a fifth to a quarter of the total REM sleep (Aserinsky, 1971; Aserinsky et al., 1985). With its relatively more active feature, phasic microstructures are innately more at risk of being rejected due to artefacts, leading to selection bias. Reducing the degree of data censoring/rejection can thus be beneficial to the generalizability of results about phasic microstructure or comparison between microstructures (Robins & Finkelstein, 2000). To achieve such purpose, further documentation into REM sleep microstructure classification is essential. As a first step, we took the initiative to identify potential challenges that may be associated with REM sleep microstructure classification in an arguably difficult setting – polysomnography recordings of patients with REM sleep behaviour disorder (RBD).

To document the difficult epochs that may be encountered during manual REM scoring (without a reliable automated tool available), we selected polysomnography recordings of patients with RBD. Due to excessive motor activities during REM sleep and frequent comorbidity with other sleep disorders (e.g. apnea), RBD polysomnography recordings are known to be challenging even for experienced sleep technicians. Through iterations of interrater concessions, we also aimed to propose solutions for the challenging epochs.

## 2 METHODS

### 2.1 Cohort description and sleep recording set‐up

**FIGURE 1:**
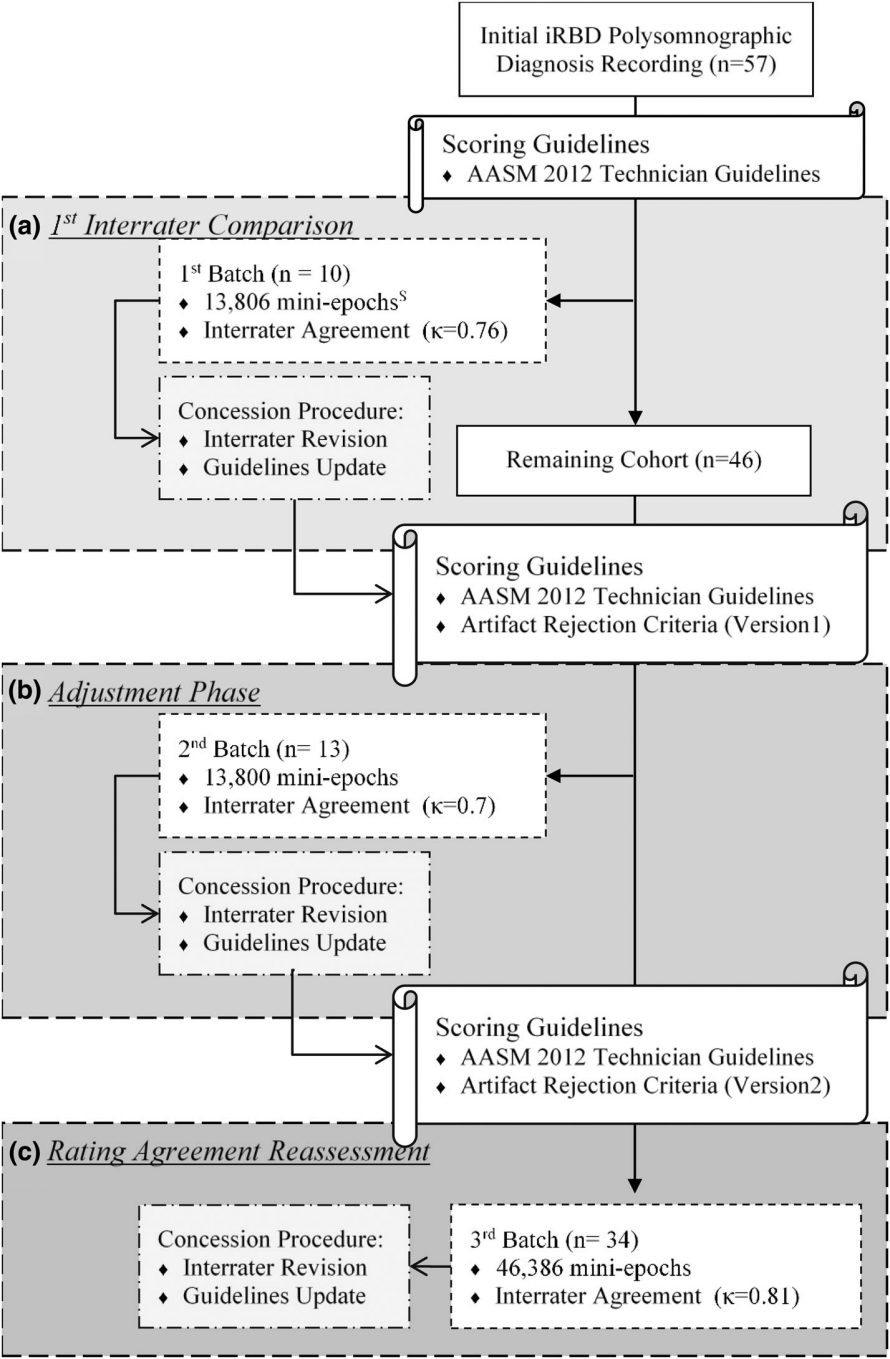
Flow chart of constructing proposed guideline.

This study included 57 polysomnography recordings randomly selected from the Montréal RBD cohort (Postuma et al., 2008; Figure 1). In brief, all five patients with RBD underwent single‐night video‐polysomnography (Grass‐Telefactor), which consisted of EEG (with standardized 10–20 system of electrode placement), EOG, electromyography (EMG) and electrocardiography (EKG), with the sampling rate at 265 Hz (sensitivity = 7 μV mm^−1^). Overnight respiration was monitored via a nasal cannula/pressure transducer. The system also encompassed a 60‐Hz anti‐hum notch filter and a 0.1/0.3–30/100‐Hz bandpass filter. Sleep staging was performed for every 30‐s epoch of all recordings by a team of sleep technicians, who performed regular concessions to ensure interrater consistency. REM sleep was defined based on the AASM manual but irrespective of the EMG signal amplitude (Iber, 2007; Rechtschaffen & Kales, 1968). It is worth noting that although spontaneous muscular activities may also occur, to a lesser extent, in healthy adults during phasic REM sleep, they generally pose little challenge to experienced raters. Together with primary goals to document challenges in REM scoring and to propose potential corresponding solutions as the primary objective (i.e. not a case–control study), no recording of disease‐free participants was included in our study.

### 2.2 EOG and display set‐up

Eye movements were measured using the standard E1‐E2 montage recommended by most current guidelines. In brief, the electrodes are placed “diagonally” around the external canthi, with E2 placed 1 cm above and 1 cm below for the E1 electrode on the contralateral side. To display REM events in the form of out‐of‐phase deflection for most types of REMs, EOG signals (i.e. E1, E2) were subtracted by the M1 mastoid electrode, A2 auriculae electrode, or an average of two auriculae electrodes (i.e. one ipsilateral pair and one contralateral pair).

Polysomnography recordings were displayed in odd‐number‐first fashion via *EDFbrowser* (an open‐source software) on a minimum screen size requirement of 13 inches (Beelen, 2008). To unify display settings, recordings were reviewed under a minimum sensitivity of 50 μV cm^−1^ (i.e. the maximum amplitude of a 1‐cm peak is 50 μV) on a 30‐s plane. Muscular artefacts were reduced via a 35‐Hz first‐degree low‐pass Butterworth filter for EOG and EEG signals as recommended by the AASM. Of note, because the purpose of the study is to assess challenges and solutions associated with REM microstructure classification, EOG signal contamination in EEG channels would not be referred to as artefacts.

### 2.3 REM detection and revision

To reduce the effect of single‐centre‐based bias, the primary execution and study process were conducted by a three‐member panel (hereon referred as the panel). These included two raters (a clinical neurologist and a sleep researcher who were previously trained at different institutions) and a local senior sleep technician, acting as an “arbitrator”. The panel first established the scoring baseline by reviewing randomly chosen segments of REM sleep recordings of participants with/without prior sleep conditions (i.e. an out‐of‐bag procedure), based on rules illustrated in the AASM sleep technician handbook (Berry et al., 2020):

“*Eye movements recorded in the EOG derivations consisting of conjugate, irregular, sharply peaked eye movements with an initial deflection usually lasting < 500 msec*”. This process was also essential to calibrate EOG epochs that were considered too artifactual to be scored as they are also an important source of interrater variance.

To allow internal testing and improvement of the solutions proposed, all recordings were randomly allocated into three consecutive batches (*n* = 10, 13 and 34) by the arbitrator. Based on the definition above, REM scoring was performed by 3‐s epochs within each 30‐s REM sleep epoch. A 3‐s epoch with at least one REM present was scored as phasic, whereas a 3‐s epoch without REM was scored as tonic (Berry et al., 2020). Undeterminable 3‐s epochs, due to the presence of artefacts, were labelled as an *artefact*. In between batches, the panel identified difficulties in REM scoring based on the interrater variance. When the opposite scoring decision persisted between the raters during the revision process, the final decision would be made by the arbitrator. Additional “rules” and potential solutions for corresponding challenges were adapted in the following batches.

### 2.4 Statistical analysis

Although the study used a qualitative‐oriented study design, we deemed it useful to assess whether interrater agreement increased between batches after applying the potential solutions for each corresponding challenge. The average interrater reliability for each scoring batch was assessed with Cohen's kappa (κ; Cohen, 1960). Effect size measure was calculated using a robust non‐parametric procedure proposed by Wilcox, which mimics Cohen's *d* (Wilcox, 2018). We compared reliability scores across batches via Kruskal–Wallis rank sum test, with the first two batches, serving as the role of control groups. Post‐hoc intergroup comparisons were performed via Dunn's test with the Benjamini–Yekutieli procedure adjusting for false discovery rate (Benjamini & Yekutieli, 2001; Dunn, 1964). The same analytical plan was used in the sensitivity analysis where all 3‐s epochs moderately‐to‐severely contaminated by artefacts were omitted. All analyses were performed in R (version 4.2.1).

### 2.5 Ethics, privacy and informed consent (method)

Written consent was obtained from all participants (or guardians of participants) in the study. Data access for the use of this study was reviewed and granted by the institutional review board (institutional reference ID: MP‐32‐2003‐1664, MP‐32‐2010‐1664 and MP‐32‐2019‐1664). An anonymization process was conducted prior to the initiation of the research conduct to protect participants' personal information and privacy.

## 3 RESULTS

### 3.1 Cohort characteristics

Of the 57 randomly selected polysomnography recordings (from the Montréal RBD cohort), batch 1 (*n* = 10) contained 13,806, 3‐s epochs (3 s), whereas batch 2 (*n* = 13) and batch 3 (*n* = 34) contained 13,800 (*n* = 13) and 46,386, 3‐s epochs, respectively.

### 3.2 REM detection: Challenges and solutions

#### 3.2.1 First batch

**FIGURE 2:**
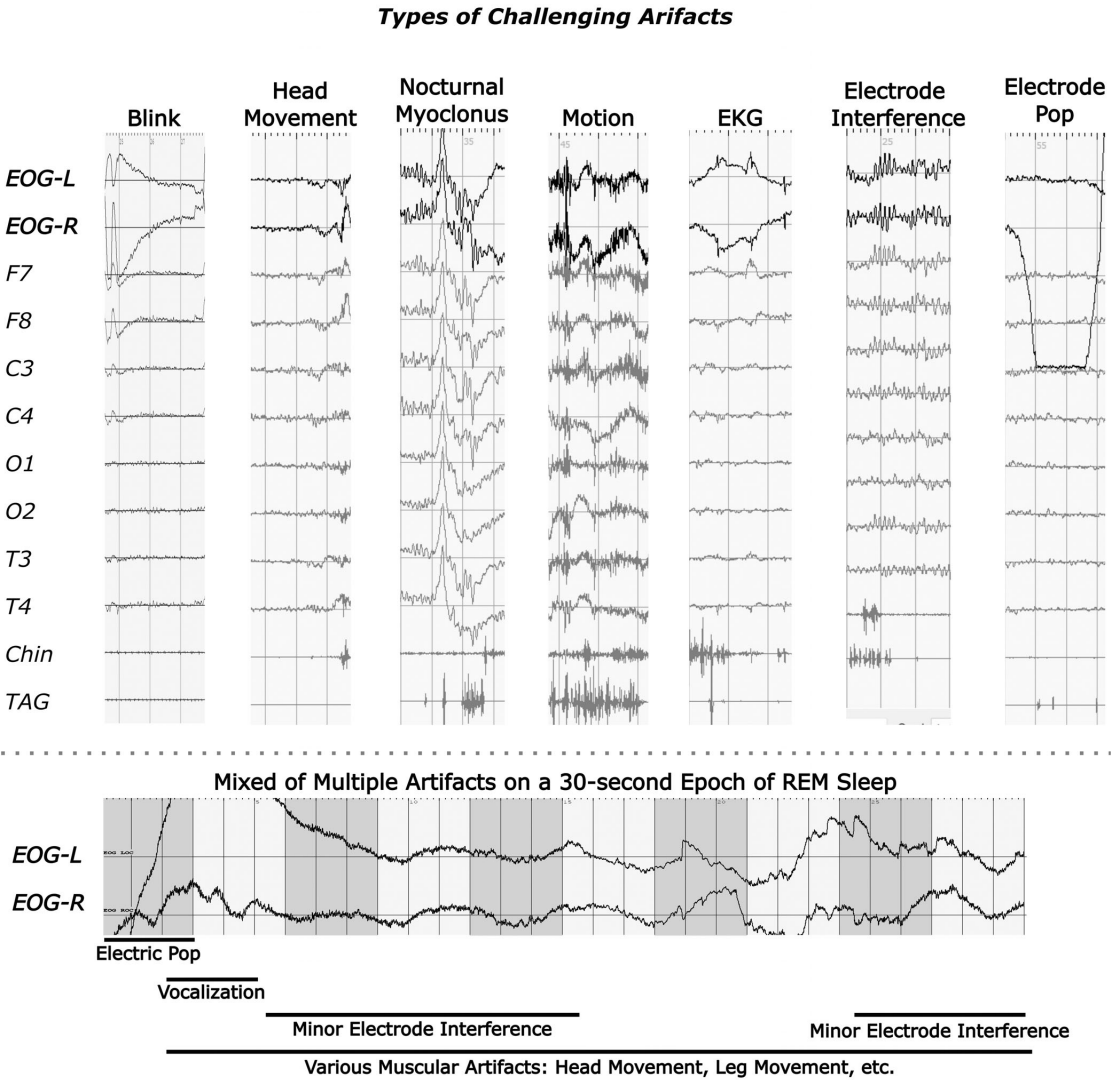
Types of challenging artefacts. Rapid eye movement (REM) detection shares many challenges known in manual sleep staging and electroencephalographic (EEG) signal analysis. Of these, several types of artefacts posed particularly difficult challenges in REM scoring. These include muscular movements (i.e. blink, head movement/turning, nocturnal myoclonus, and motion/body movement), electrocardiography (EKG) signal contamination and electrode artefacts (electrode interference and electrode pop). A realistic view of multiple artefact interferences was illustrated in the lower panel. Each horizontal bar indicated the duration of an artefact interference.

Based solely on the current AASM guidelines, the primary source of interrater variation was discordance on raters' decision on rejecting an artefact‐contaminated epoch (i.e. to what degree a REM epoch should be rejected due to an artefact) during the first interrater revision. Besides common challenges involving the co‐presence of multiple artefact subtypes, several artefacts were particularly prevalent among the non‐agreeing epochs (Figure 2). These included moderate‐to‐severe distortion in signals due to muscular movements (e.g. blink, head turn, movement at extremities and bruxism), EKG signal contamination (i.e. ventricle depolarization in the form of QRS‐complex) and electrode artefacts (e.g. electrode pop and reference artefact). It is worth noting that challenges often arose from the co‐presence of several types of artefacts but seldom individually, as illustrated in the lower panel of Figure 2.

**FIGURE 3:**
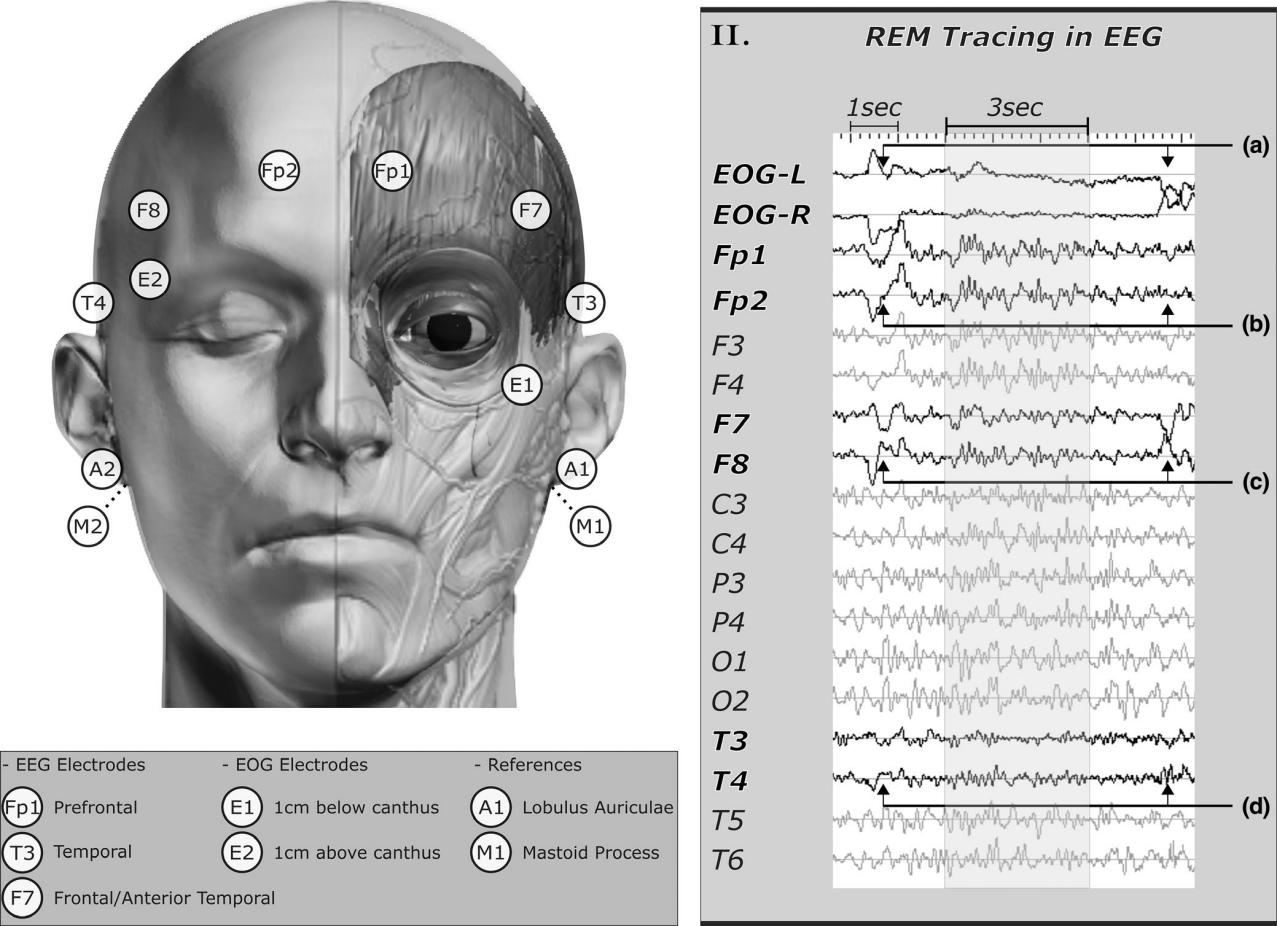
Electrooculogram (EOG)‐artefacts in specific electroencephalographic (EEG) electrodes serve as potential proxies for rapid eye movement (REM) detection. Electrodes of both EOG and EEG were placed based on the AASM recommended guidelines. (II‐a) REMs were displayed in the form of anti‐phase deflection using E1‐A2 and E2‐A2 derivates with amplitude sensitivity set at 200 μV cm^−1^. (II‐b–d) REM traces can frequently be found in Fp1/2, F7/8 and T3/4 due to their proximity to the corneal‐retinal dipole, as displayed with amplitude sensitivity set at 50 μV cm^−1^. (II‐b) At Fp1/2, REM traces are frequently shown as in‐phase deflections for oblique or vertical eye movement. (II‐c) REM traces in F7/8 generally share a similar deflection shape as seen in EOG. (II‐d) Similar but much weaker deflection can also be found infrequently in T3/4.

To reduce interrater variance, artefact‐contaminated mini‐epochs with clear presence of REM events in EOG tracing or via the aid of EEG electrode proxies (i.e. EOG‐artefacts) were henceforth labelled as REM (Figure 3II‐b–d). Whereas muscular and cardiac artefacts typically induce scalp‐wise distortion in the EEG signals under visual inspection (Figure 2), the shadow‐like REM traces in EEG signals would appear primarily in specific channels (i.e. Fp1/2, E7/8 and T3/4; Figure 3II‐b–d). Of the three pairs of proxies, F7/8 were generally better at preserving REM morphology (e.g. deflection directions and signal amplitude) than Fp1/2 and T3/4. As a “background noise”, the amplitude of these shadow‐like REM traces would decrease as the distance to EOG electrodes increases. As such, both raters also found it helpful to unravel REM masked by artefacts by adjusting the signal display amplitude sensitivity (e.g. increasing from 50 μV cm^−1^ to 200 μV cm^−1^).

#### 3.2.2 Adjusting phase

Besides adapting to the solutions formed during the prior concession, raters were also instructed to take notes of other potential challenges during REM scoring and assessed potential corresponding solutions. Among the 13 “new” recordings, several challenges emerged from non‐agreeing mini‐epochs. Specifically, these included the following.

**FIGURE 4:**
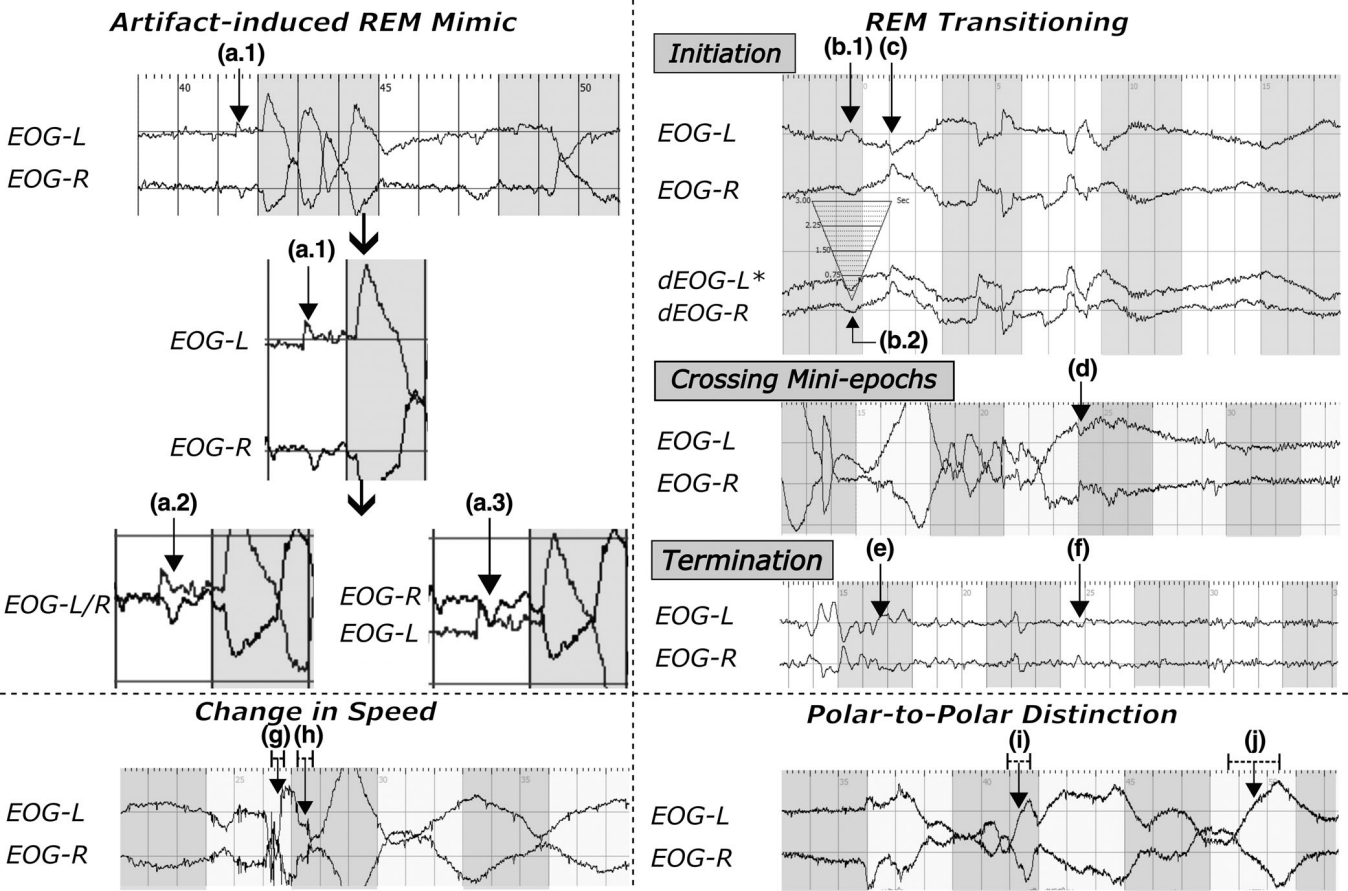
Artefact‐induced rapid eye movement (REM) mimics and phenomenology of REM. All electrooculographic (EOG) signals are displayed at an amplitude sensitivity of 200 μV cm^−1^ to fit the display window. Signals were segmented into a series of 3‐s epochs with different background colours between the adjacent mini‐epochs (i.e. grey and white). The top‐left corner displayed an example of artefact‐induced REM mimics when displayed on a screen with (a.1) default setting, (a.2) two EOG‐signals aligned for their baselines, and (a.3) two EOG‐signals reversed in their positions. EOG signals displayed at a.2, a.3 and the second a.1 were zoom‐in screenshots. An additional EOG duplicate can be useful to determine if a deflection is a mimic of artefact(s). The top‐right corner displayed common challenges that occur during a series of REM events (i.e. phasic REM sleep) and transitioning between the two REM sleep microstructures. (b.1) An ambiguous REM event with the initial deflection of 0.5 s during the initiation of a phasic REM sleep episode. (b.2) The conjugation of the same ambiguous REM event in duplicated signals (dEOG) with the left EOG signal inversed. (c) The first clear REM event at the initiation of a phasic episode. *Asterisk indicated an inversed signal. The label (d) illustrated a REM event crossing two mini‐epochs. The last panel illustrated a potential challenge at the end of a phasic REM sleep episode, with (e) indicating the last clear REM event and (f) for an ambiguous REM occurring a few seconds after the last clear REM. Because the goal of REM scoring was to determine steady phasic REM microstructure, the ambiguous REM event—b.1, adjacent to a clear REM event, at the initiation would be considered as REM, but not at (f). An example of changes in eye movement speed during a REM event was displayed at the bottom left. The embedded labels illustrated (g) a REM event and (h) slowing in eye movement speed halfway through. For the latter, we recommended it be treated as two separate REM events separated by the slowing at (h). The bottom‐right corner showed REM events across two poles (e.g. from “looking” towards left to “looking” at right). The labels pointed at (i) a polar‐to‐polar REM event and (j) a polar‐to‐polar slow eye movement (SEM).

1. Artefact‐induced REM mimics (Figure 4a.1–3): these types of artefacts typically presented with a few microseconds of delay in the initial deflection. To avoid misclassifying an artefact‐induced REM mimic, raters proposed to duplicate EOG channels (Figure 4a.2–3) to evaluate the delay. Because a delay is apparent, A1 would not be considered as REM.

2. REM sleep microstructure transitioning (Figure 4b.1–2,c,e,f): another potential source of interrater variance arose from the difference in scoring decisions made at the beginning and the end of a phasic REM microstructure. Morphologically, REM might be slightly rounder than the typical REM. For instance, B.1 illustrated an out‐of‐phase conjugate deflection with less than 0.5 s duration for the initial deflection while roundish in shape under 100 μV cm^−1^ display sensitivity. Of note is that the same ambiguous REM event might appear pointy when increasing the display sensitivity to 50 μV cm^−1^. For these, we propose that any ambiguous REM event presenting within an adjacent 3‐s mini‐epoch prior/after the nearest apparent REM would, hereafter, be considered as REM to reduce the interrater variance for mini‐epochs during the phasic/tonic transitioning. In time domain (label F), some miniature conjugate deflections resembling that of labels C might not be immediately adjacent to a series of train‐like REM events typically during a phasic REM episode. These “distanced” events would not be scored as REMs.

3. REM events crossing two mini‐epochs (Figure 4d): due to the constraint from the pre‐defined time window for each epoch, REM events could occasionally cross over from one epoch to the next in the middle of a phasic episode (Figure 4d). In such a scenario, raters sometimes made different scoring decisions for the adjacent epoch with fewer REM crossing events. To unify the scoring rules, such a REM event would be shared by both unless one contains less than a third of the REM.

4. Sudden change in speed during deflection (Figure 4g,h): when sudden changes in the eye movement speed occur halfway through a deflection (Figure 4h), the deflection would be treated as two distinct REM events separated by a short deceleration period. Raters would also factor this rule into consideration when such a type of deflection crosses two mini‐epochs.

5. REM across two poles (Figure 4i,j): this type of event might occur during the middle of a train‐like REM event towards the end of the sleep period (Figure 4i). It appeared seemingly as if the sleeper is looking from left to right. When this happens, raters would make a decision using half of the period spent during the initial deflection. If half of the initial deflection duration was less than 0.5 s, such eye movement would be considered as a REM (Figure 4i). If it is longer than 0.5 s as in label J it would not be identified as a REM.

**FIGURE 5:**
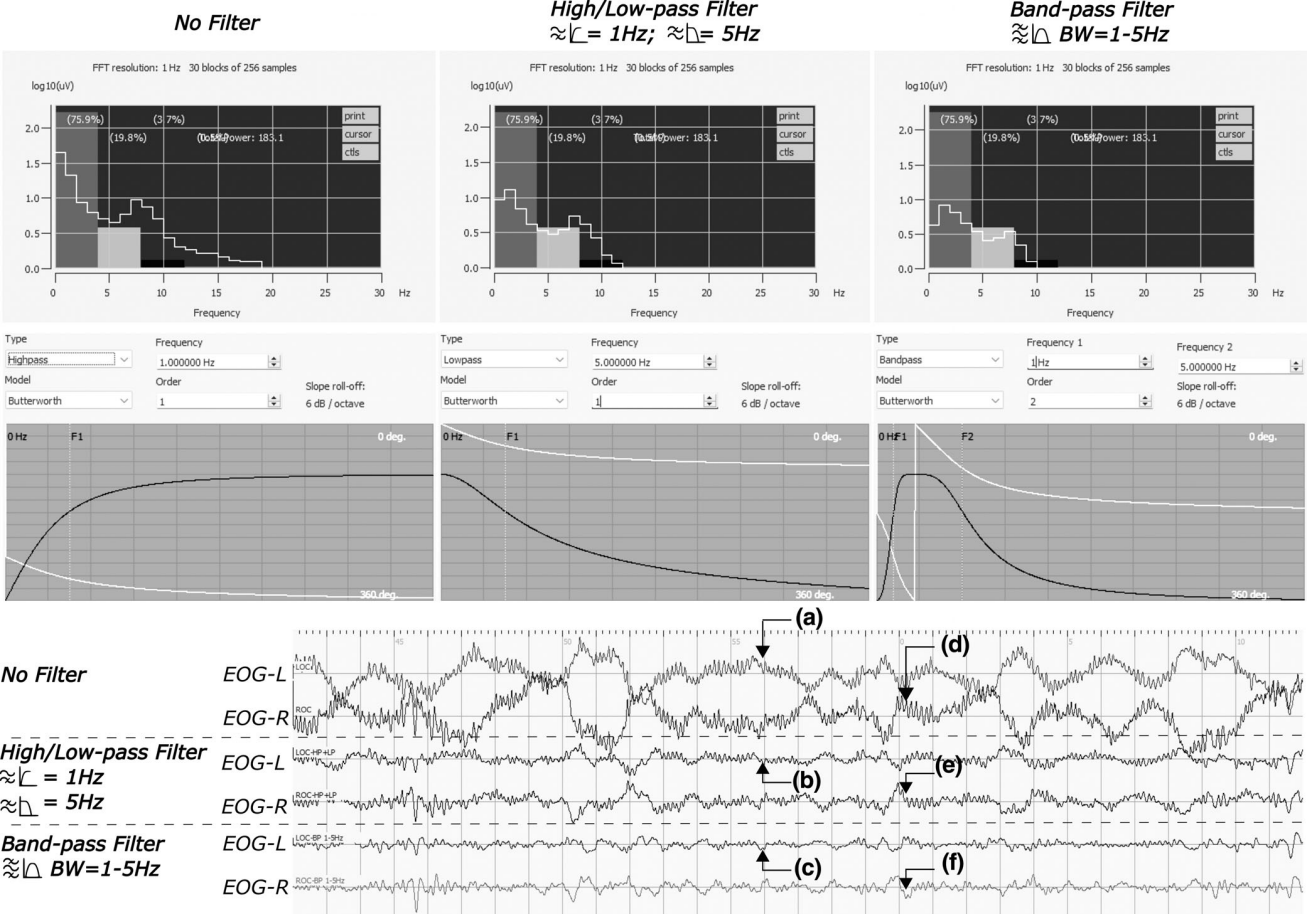
Filter selections—serial high‐/low‐pass filter versus bandpass filter. Electrooculographic (EOG) signals were displayed in a window of 30 s at a minimum amplitude sensitivity of 100 μV cm^−1^. To illustrate the effect of the filters, two sets of EOG‐pair duplicates were generated. For the serial high‐/low‐pass filter, a combination of a first‐degree, 1‐Hz high‐pass filter and a first‐degree, 5‐Hz low‐pass filter was used. The same frequency range was also applied to the second‐degree bandpass filter. Butterworth algorithm was used for both filters. As shown, both filters were able to suppress noise present at high frequencies (> 10 Hz, top‐left window), thus revealing the EOG signals underneath for all labels on the left (a–c). On the right side, a peak unseen in the original non‐filtered signals (d) or under an active serial high‐/low‐pass filter (e), was shown in bandpass filters (f).

**FIGURE 6:**
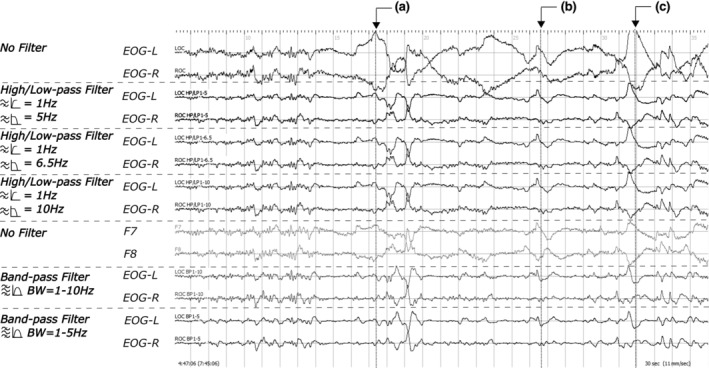
Frequency selections for serial high‐/low‐pass filter and bandpass filter. Several frequency bands were assessed in both the serial high‐/low‐pass filter and the bandpass filter with the lower bound set at 1 Hz. On the contrary, the upper bound varies among 5, 6.5 and 10 Hz. The effectiveness of noise suppression was evaluated via power spectrum estimates, as shown in Figure 5. Rapid eye movement (REM) peaks could be found (a) aligning along the left line, but (c) shifting across all settings along the right line with most shifts for those using a bandpass filter. (b) Both filters were not able to fully restore REM under the influence of the pressure‐artefact at the right electrooculogram (EOG) lead due to the sleeping position.

Besides the above solutions, we also assessed the use of second‐degree serial high/low‐pass filter and bandpass filter at several frequency bands (1–5, 1–6.5, 1–10 Hz; Figures 5 and 6) as aids in confirming REM when masked by artefacts. Both serial high/low‐pass and bandpass filters showed usefulness in suppressing artefacts and unveiling REM (Figure 5a–c), although a peak, unseen in unfiltered EOG channels (Figure 5d) or in serial pass filters (Figure 5e), appeared in signals passed through a bandpass filter (Figure 5f). When adjusting frequency bands, deflections were preserved across all settings (Figure 6a). Two pitfalls were noted with the use of filters regardless of frequency band settings. First, filters may not be adequate for segments with electrode‐related‐artefact on a signal channel compared with REM preserved as an EOG‐artefact in F7/8 (Figure 4b). In addition, because an EOG recording would contain signals with a broad spectrum of frequencies, filtering could potentially lead to a time‐advanced‐like shift (Figures 6c and S1). This type of phenomenon may be especially more pronounced when slowing occurs at the end of a deflection (Figure 6c).

#### 3.2.3 Rating agreement reassessment

**FIGURE 7:**
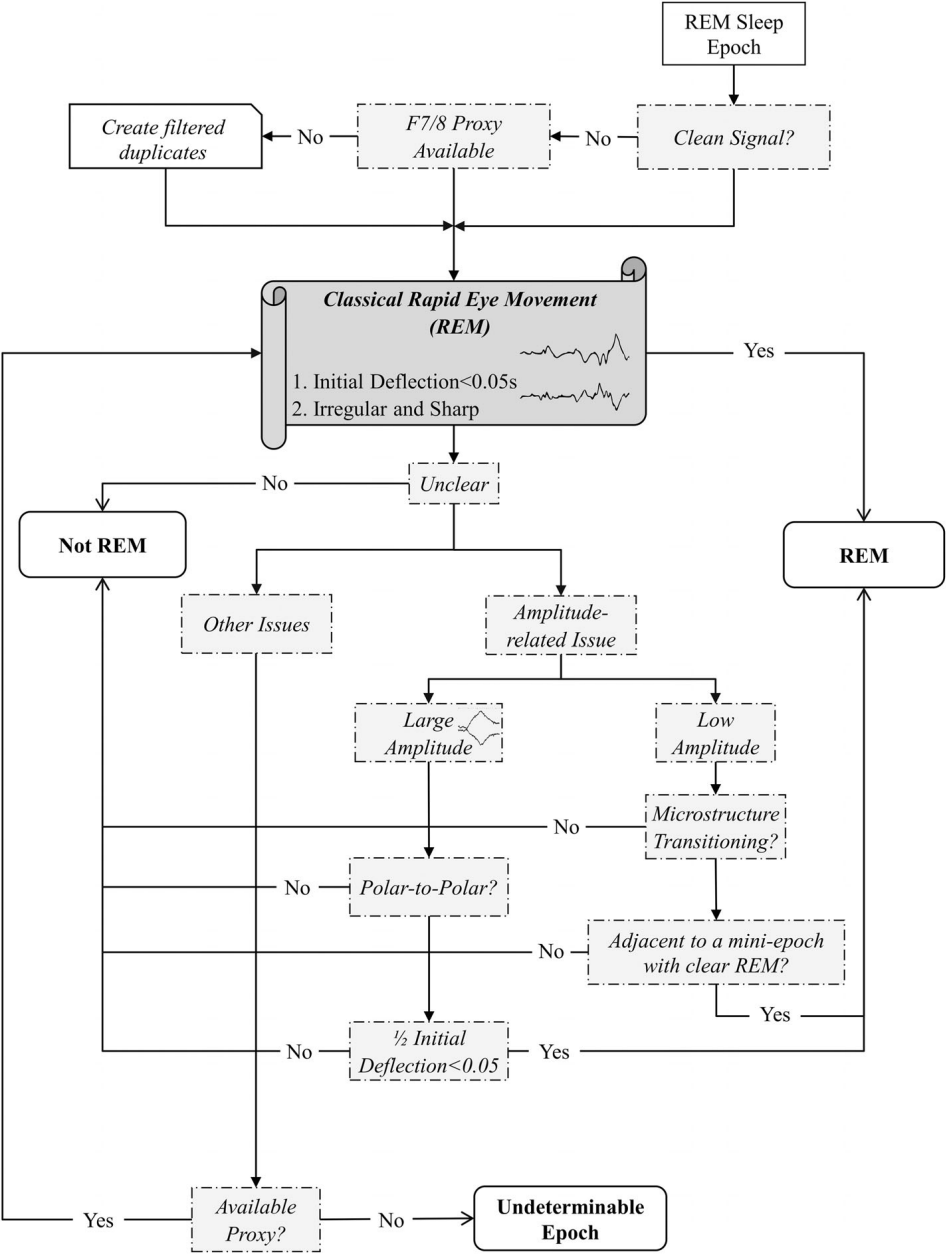
Schematic graph for rapid eye movement (REM) detection. The decision‐making process for REM detection stems from our study.

Pooling together the influences of artefacts and the proposed solutions/guidance above, a triage‐like schematic graph was formed aiming to reduce interrater variance for REM detection (Figure 7). In brief, under the influence of artefacts, raters could first refer to F7/8 (Figure S2a,b) or a 1–5‐Hz serial high/low‐pass filtered EOG‐pair duplicate (if the former is unavailable). When in doubt, raters would refer to the proposed guidance provided based on the amplitude/shape of an eye movement or other additional proxies. During REM sleep microstructure transitioning, only ambiguous REM events immediately adjacent to a clear REM event would be labelled as REM. An epoch would be labelled as an *artefact* if it is undeterminable due to being heavily distorted by artefacts (e.g. reference artefact in Figure S2). Nonetheless, artefact rejection would be contingent on the performance of solutions proposed. For instance, under the influence of REM sleep without atonia, raters would consider the first six and the last 3‐s epochs of Figure S3 as tonic microstructures (ME1–6, 10), while simultaneous out‐of‐phase deflections from the seventh to the ninth epochs could be confirmed via filtered EOG‐duplicates alone (ME7–9).

**FIGURE 8:**
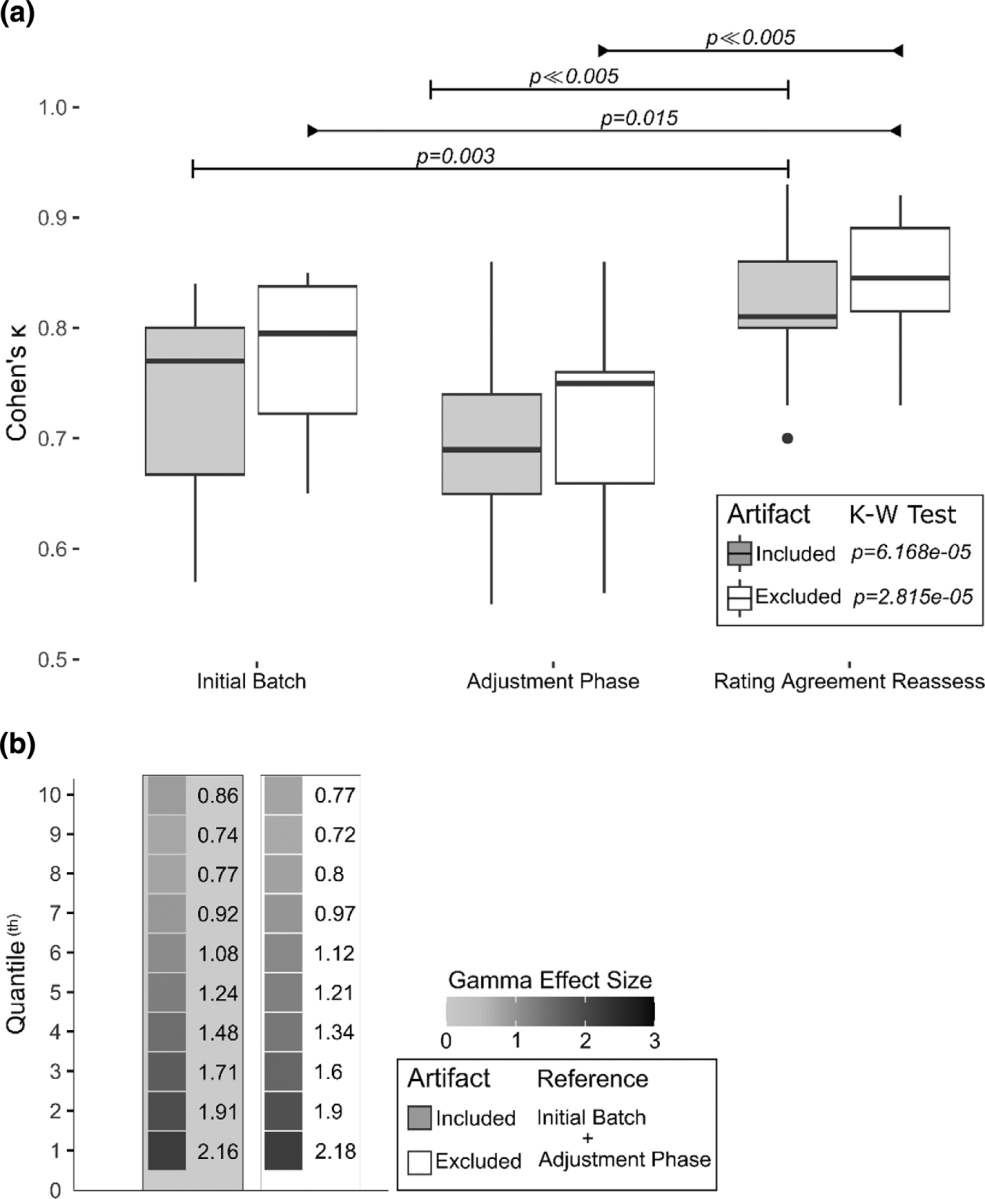
Interrater agreement comparison. Interrater agreements were evaluated for each recording via Cohen's kappa. (a) Overall changes in scores were assessed using the Kruskal–Wallis rank sum test (i.e. K‐W test; grey: with artefacts included; white: with artefacts excluded). An intergroup comparison was performed using Dunn's test (Dunn, 1964). Probability values displayed were adjusted for false discovery rate (Benjamini & Yekutieli, 2001). (b) To illustrate the change of interrater agreement before the use of the proposed solutions/guidance, we pooled together the results from the initial batch (i.e. the first batch) and during the adjustment phase (i.e. the second batch) as the reference group. The left column in grey indicated that artefacts were included (which were otherwise excluded in the right column in white). Changes in interrater agreement levels, after the use of the proposed solutions/guidance, were presented in non‐parametric effect sizes, a Cohen's *d*‐consistent analogue (Wilcox, 2018). Sensitivity analysis was carried out for the subsets after omitting artefact‐contaminated mini‐epochs.

Using 34 additional recordings, both raters reassessed the effectiveness of the proposed solutions/guidance for reducing interrater variance (Figure 7). When systematically applying the proposed solutions above, interrater variance was significantly lower during the reassessment (mean ± SD = 0.83 ± 0.05, *p* < 0.005) than both prior batches (first batch = 0.76 ± 0.08, second batch = 0.72.0 ± 0.09) after correcting for false discovery rate (Figure 8a). Of the 34 recordings, 27 received kappa scores above 0.8 (i.e. almost perfect agreement). In addition, most quantiles showed strong improvement (i.e. effect size > 0.8) when compared with the pooled reference group (i.e. the prior batches). The results remained similar after removing all mini‐epochs with moderate‐to‐heavy contamination of artefacts (Figure 8b).

## 4 DISCUSSION

Capitalizing on the complex nature of sleep comorbidities involved in RBD, we were able to identify potential challenges (e.g. artefacts) involved in manual REM scoring for classifying REM sleep microstructures using 73,992, 3‐s mini‐epochs. Besides artefacts of electrical interferences and cardio‐/myogenic source, interrater variances commonly present during the transitioning of REM sleep microstructures, changes in eye movement speed and REM events crossing two mini‐epochs, and two poles (e.g. horizontal eye movements from left to right). Summarizing the potential solutions/guidance assessed, we provide a schematic graph for manual REM scoring, found effective in improving interrater agreement.

### 4.1 Artefacts in EOG and potential solutions

In 1953, Aserinsky had already noted several artefacts present in EOG, many of which are also frequent challenges in sleep staging and EEG signal processing (Aserinsky & Kleitman, 1953). Although multiple types of artefacts often coincide, we found artefacts of myogenic or electrical sources to be more challenging than that of cardiorespiratory events. One such reason could be because respiration (15.2 ± 3.0 per min) mainly affects the low‐frequency domain of SEM (0.2–0.5 Hz; Gutierrez et al., 2016). Sleep apnea also seldom affects REM detection as it tends to promote arousal or transition to non‐REM sleep (Eckert & Younes, 2013). At around the lower bound of REM frequency band (~1 Hz), EKG‐artefact can often be seen along EOG signals in the form of miniature peaks resembling the QRS complex (Figures 2 and S4). In general, we found little challenge when EKG‐contamination was the sole source of artefacts in EOG signals. One possible exception was when an EKG‐artefact occasionally distorted an ongoing deflection, masking the peak of a REM (Figure S4‐a). Such influence could often be resolved with the use of additional references/proxies, as suggested in the lower half of the schematic graph (Figure 7).

Both muscular movements (e.g. eyelid, jaw, head and limbs) and electrode/electrical interferences posed difficult challenges, as they frequently mask REMs (Figures 5a and 6b). As such, raters would often first examine the usability of F7/8 proxies to decide if filtered EOG duplicates should be generated as shown at the top of the proposed schematic graph (Figure 7). Muscular activities can also be noted during phasic REM sleep in healthy adults, although it is particularly common among patients with RBD due to REM‐sleep‐without‐atonia (Frauscher et al., 2012; Simor et al., 2020). Similar to EKG artefacts, proxy electrodes (e.g. F7/8) can often be helpful with the presence of muscular artefacts. In certain cases where muscular artefacts distorted both EOG and EEG channels, raters may also consider using a superimposed‐EOG‐duplicate (Figure 4a.2) or a single‐channel‐inversed‐EOG‐duplicate (Figure 5b.2) to rule out misaligned artefactual signals. It is worth noting that these two techniques may be less effective in the presence of electrode/electrical interferences (Figures 5 and S2‐d) as they often require the use of filtering techniques as in EEG analysis (Widmann & Schröger, 2012). Additional filtering is thus recommended when generating EOG duplicates (Figure 7).

### 4.2 REM sleep microstructures: Biphasic versus non‐binary continuum

Besides artefacts, a large portion of interrater variance came from the transitioning between REM sleep microstructures (Figure 4b–f). These two microstructures, which can be distinguished by the presence of REM (i.e. phasic – with REM; tonic – without REM; Moruzzi, 1963; Simor et al., 2020), are believed to have a flip‐flop relationship strongly associated with the elicit of ponto‐geniculo‐occipital (PGO) waves (Fernández‐Mendoza et al., 2009; Gott et al., 2017). Unlike a flip‐switch, we frequently noticed some rapid but small eye saccade REMs, occurring in a timeframe of a few mini‐epochs before/after a typical known REM saccade during the transitioning between microstructures (Figure 4b,c). At the initiation of a phasic microstructure, these eye movements often share similar descriptions as to microsaccades, which are 1–2‐Hz low‐amplitude jerky eye movements with 1° of arc frequently elicited after the onset of PGO waves (Coakley et al., 1979; Ermis et al., 2010). Nonetheless, because EOG is not sensitive enough to capture these small train‐like movements, it is more likely that these are not initial REMs but microsaccades followed by PGO waves. On the temporal domain, these ambiguous REM events coincide with the continuum transitioning hypothesis suggested by Bueno‐Junior, because they were not always adjacent to the nearest REM event easily identified (Bueno‐Junior et al., 2022; Figure 4e,f). As such, they posed additional challenges that resulted in increases in interrater variance. Although the proposed criterion requiring a clear REM adjacent may reduce interrater variance resulting from ambiguous REMs (Figure 7), this arbitrary method was made primarily based on the flip‐flop mechanism for microstructure transitioning. Future studies will be needed to develop guidelines to facilitate interrater agreements specifically for this issue.

### 4.3 Potential solutions for REM scoring

#### 4.3.1 Frontal scalp EEG leads and eye movements

The presence of eye movements in EEG signals (i.e. EOG‐artefacts) has also been known since the initial introduction of REM (Aserinsky & Kleitman, 1953). Of the electrodes along the 10–20 system, REM‐artefacts are particularly common among prefrontal (Fp1/2; Figure 3II‐b) and frontal/anterior temporal (F7/8; Figure 3II‐c) leads with occasional traces at medial temporal electrodes (T3/4; Figure 3II‐d; Ai et al., 2016; Aserinsky & Kleitman, 1953; Aserinsky & Kleitman, 1955; Plöchl et al., 2012). Of the two former electrode pairs, the F7/8 proxy can generally capture both horizontal and vertical components of an eye movement, whereas prefrontal leads reflect predominantly the vertical component (Ai et al., 2016). As such, this makes F7/8 a preferable proxy for REM detection when the EOG signals are ambiguous or compromised (Figure 7). Although the use of F7/8 was also recommended by Aserinsky (Aserinsky & Kleitman, 1953; Aserinsky & Kleitman, 1955), this method could be overpowered by certain artefacts, especially that of an electrode/electric source (Figure S2‐d). In addition, it is worth re‐emphasizing that the current recommended EOG montage alone cannot determine the eye movement direction, as observed (Berry et al., 2020; Figure 3II).

#### 4.3.2 Energy decay and F7/8 proxy

The shadow‐like REM traces in F7/8 electrodes are about a quarter to half of their original amplitudes in EOG (with a mono‐polar system; Figure 3II‐a,b). Therefore, raters may consider adjusting the amplitude sensitivity displayed accordingly. Interestingly, a similar note was made by Aserinsky, where it was suggested as a result of the electrode placement designs (i.e. bipolar versus monopolar; Aserinsky & Kleitman, 1953). Because both EOG and EEG placements used in our study were of the monopolar design, the shadow‐like REM traces should have shared similar amplitudes as the originals. One possible alternative reason for this phenomenon is that the amplitude is negatively correlated with the distance from the signal source (i.e. retinal dipole). As seen in Figure 3, the amplitude of REM traces seems much lower in the T3/4 pair (II‐d) than in F7/8 (II‐c). Similar phenomena were also shown in several studies assessing EOG‐artefacts across EEG signals (Ai et al., 2016; Plöchl et al., 2012). Due to the energy decay, the F7/8 proxy would likely be less sensitive for low‐amplitude REM that commonly occurs at the beginning and the end of each phasic REM sleep period (Figure 4b,f).

#### 4.3.3 Serial high‐/low‐pass filters

In our study, the use of F7/8 proxy was often overpowered by artefacts. To unveil the EOG signals (sensor range = 0.05–30 Hz), we assessed the use of low‐ and high‐pass filters, both individually and in a series (Figures 4 and S‐1,2). The major distinction between a SEM and REM is the frequency, where the former generally occupies frequencies below 1 Hz (commonly found in the range of 0.2–0.5 Hz; Virkkala et al., 2007) and above 1 Hz for the latter (Berry et al., 2020). In a recent RBD study, the Copenhagen team estimated that the true range of REM activities lies between 1 and 10 Hz, whereas the 10–30‐Hz band contains nearby muscular activities (e.g. orbicularis oculi; Kempfner et al., 2011). Although the 10‐Hz cut‐off was made based on the common recommendation for EMG filter setting, their estimate of REM cohered with Gopal's finding (Gopal & Haddad, 1981). Using EOG recordings of sleeping infants, Gopal noted that 95% of signal power lies within 6.5 Hz.

Interestingly, we found no clear difference in frequency settings visually (i.e. 1–5, 6.5, 10 Hz) for the serial high‐/low‐pass filter with a second‐degree order (Figure 6). This was likely due to the leakage from the filter. In fact, the frequency leakage of a low‐order filter would be larger than that of a high‐order filter and thus preserves the shape of the oscillation in the time domain. Instead of abruptly suppressing signals like notch filters, low‐order pass filters suppress noises in gradient roll‐off frequency, where it is weaker for nearby frequencies. The main difference came from the choice between the serial high‐/low‐pass filter and bandpass filter. Although both filters technically share the same purpose (Figure 5a–c), these two filter designs are not algebraically equivalent (Ozenbaugh & Pullen, 2017). In our study, changes in the shape of a deflection tended to be more abrupt for bandpass filter (Figures 5f and 6c). This could occasionally create false REM events in the following epoch (Figure 5f), thus making the serial high‐/low‐pass filter more reliable. Nonetheless, we would caution against the practice of performing REM detection on filtered signals alone, because this could create potential bias. For instance, in Figure S1, the end of the initial deflection (i.e. the maximum value) was suppressed due to slowing in eye movement speed. Alternatively, raters may consider generating two extra EOG duplicates each with a high‐ or low‐pass filter.

### 4.4 Data censoring and open science

One of the motivations behind our study is to explore effective solutions to reduce the biases from data censoring. While such a notion has not been well‐known beyond epidemiology and biostatistics, data censoring is known to introduce biases, which persist even in case–control studies (Robins & Finkelstein, 2000). With the use of the proposed solutions, we demonstrated its ability to identify the otherwise unclear REM events (Figure S3 ME7–9). Subsequently, the proposed open‐source‐tool‐based solutions provide aid in preserving more usable epochs for various biosignal analyses thus reducing the effect of data censoring. Nonetheless, as the first study providing detailed technical documentation on REM sleep microstructure classification, external validation is welcome and needed.

### 4.5 Strengths and limitations

Several limitations in our study should be noted. First, we included only the E1‐E2 montages commonly recommended by modern guidelines (Figure 3). Unlike the multiple bipolar approach, employed in early studies, the E1‐E2 montage was designed to assess vertical and horizontal eye movements simultaneously (Berry et al., 2020; Feinberg et al., 1969). On the other hand, the E1‐E2 montage risks missing small REM events, thus leading to a lower REM count (especially among younger adults), as noted by Feinberg et al. (1969). Because patients with RBD are generally older, the influence on our results might be negligible. Nonetheless, the challenges and the corresponding solutions presented in this report may be limited for other alternative EOG montages. We also recognized the limitation in identifications of blinking artefacts with the E1‐E2 montage. On the vertical plane, blinking artefacts could be easily identified by their unidirectional features. Whereas with the E1‐E2 montage, the blinking artefacts can be challenging due to inability to determine eye movement direction (Berry et al., 2020; López et al., 2016; Rechtschaffen & Kales, 1968). One possible solution to this is to examine the simultaneous activities seen in EEG leads as shown in the technician handbook. Third, the proposed scoring techniques (Figure 7) were only systematically applied by raters during the final batch. Although this would likely explain the lack of difference in performances between the initial batch and the adjusting phase (Figure 8a), we could not rule out the positive impact of discussions during interrater revisions. Fourth, we also recognized that the proxy electrodes proposed (i.e. F7/8) are often excluded from standard clinical practice. While REM artefacts may theoretically be observed on electrodes across the scalp (Ai et al., 2016), F7/8 remained preferable over the electrodes commonly used in clinical laboratories (i.e. F3/4, C3/4, O1/2) due to its consistency in displaying REM events (Figure 3‐II). As such, the inclusion of F7/8 may be considered for future clinical practice, especially when excessive muscular activities are expected. Finally, because the study was designed primarily to document challenges and corresponding solutions to REM scoring, the improvement in REM scoring could serve only as a support to the potential effectiveness of the proposed scoring methods (Figure 7). Future multi‐rater/centre validation will be required to assess their effectiveness for reducing interrater variance.

On the other hand, this study has several strengths. The primary one was the simultaneous REM scoring involving 57 RBD polysomnography, 73,992 mini‐epochs (3 s) by two raters. To the best of our knowledge, this is one of the largest studies on REM detection by multiple raters, and the first methodological report on the identification of associated artefacts and challenges. Second, to avoid single‐rater and‐centre‐bias, no one rater or author was involved in both sleep staging and REM sleep microstructure classification of any recordings included. In addition, all members of the panel (i.e. two raters and the arbitrator) were all trained at different institutions. Such a multi‐rater approach is uncommon even in most quantitative studies given the long recording time. Nonetheless, even with the multi‐rater strength, we would re‐emphasize the essential need for future validation to assess the generalizability of the proposed guidelines/solutions. Third, by capitalizing on the complex nature of sleep disorder comorbidities in RBD, we were able to document various potential challenges and assess the proposed solutions (Schenck et al., 2019). In addition, because spontaneous muscular activities may occasionally occur during normal phasic REM sleep, the proposed scoring techniques (Figure 7) may have some potential for general usage in REM microstructure research and training. To translate such a procedure into clinical practice, we will require further understanding into the distinct neurophysiological mechanisms underlying REM sleep microstructures. Fourth, both raters were blinded for the polysomnography recording selection process and patients' clinical histories/outcomes throughout the study. This allowed us to reduce potential bias that could have been introduced during the sampling and improve the reliability of the results. Finally, to account for diversity, equity and inclusion, we intentionally designed the study to rely on an open‐source tool (i.e. EDFbrowser). To facilitate the use of our proposed methods, we also provided three EDFbrowser setting files with the proposed scoring techniques. With these files, users can switch between settings via keyboard shortcuts without the need to create duplicated signals or move channels via a computer mouse. A brief tutorial regarding the implementation (including EDFbrowser installation and assigning keyboard shortcuts) can be found in the supplementary material.

## 5 CONCLUSIONS

Our study illustrated potential solutions and guidance for challenges that may be encountered during REM sleep microstructure classification. The development of detailed REM detection guidelines is needed.

## AUTHOR CONTRIBUTIONS

**C. William Yao:** Conceptualization; investigation; methodology; data curation; visualization; writing – original draft; writing – review and editing; formal analysis. **Giuseppe Fiamingo:** Conceptualization; methodology; writing – review and editing; writing – original draft; investigation. **Karine Lacourse:** Methodology; software; data curation; formal analysis; writing – review and editing; validation. **Sonia Frenette:** Data curation; resources; validation; conceptualization; methodology; writing – review and editing; project administration. **Ronald B. Postuma:** Data curation; writing – review and editing. **Jacques Y. Montplaisir:** Data curation; writing – review and editing. **Jean‐Marc Lina:** Conceptualization; methodology; funding acquisition; validation; investigation; writing – review and editing; writing – original draft; resources; supervision; project administration; data curation. **Julie Carrier:** Data curation; supervision; resources; project administration; validation; methodology; writing – review and editing; writing – original draft; funding acquisition; investigation; conceptualization.

## FUNDING INFORMATION

This study was funded by NSERC‐CREATE grant, Fonds de la Recherche en Santé, The Parkinson Society of Canada, Weston‐Garfield Foundation, Michael J. Fox Foundation, and Webster Foundation.

## CONFLICT OF INTEREST STATEMENT

All authors report no non‐financial support from outside of the submitted work. C. William Yao, Giuseppe Fiamingo, Karine Lacourse, Sonia Frenette Jacques Y. Montplaisir and Jean‐Marc Lina report no financial disclosures. Ronald B. Postuma reports personal fees from Biotie, Roche/Prothena, Teva Neurosciences, Novartis Canada, Biogen, Boehringer Ingelheim, Theranexus, and GE HealthCare, outside the submitted work. Julie Carrier reports grants from Eisai.

## DECLARATION OF APPROVAL FOR SUBMISSION

The submitted manuscript has been reviewed and approved by all authors.

## Supporting information


**DATA S1.** Supporting Information.

## Data Availability

The data that support the findings of this study are available on request from the corresponding author. The data are not publicly available due to privacy or ethical restrictions.
