# Supplementary material for: Technical challenges in REM sleep microstructure classification: A study of patients with REM sleep behaviour disorder
Source: J Sleep Res. 2024 Apr 12;33(6):e14208. doi: 10.1111/jsr.14208 (PMC11597017; doi:10.1111/jsr.14208)
Supplement: Supplementary file 1 — DATA S1. Supporting Information. [file JSR-33-e14208-s001.docx]

***Technical Challenges in REM Sleep Microstructure Classification: A Study of Patients with REM Sleep Behavior Disorder***

^$,1,2^C. William Yao, *PhD*; ^$,2,3^Giuseppe Fiamingo, *M.D.*; ^2^Karine Lacourse, *M.Sc.*; ^1,2^Sonia Frenette, *B.Sc.*; ^2,4,5^Ronald B. Postuma, *M.D. M.Sc.*; ^2,6^Jacques Y. Montplaisir, *M.D. PhD*; ^*2,4,5^Jean-Marc Lina, *PhD*; ^*1,2^Julie Carrier, *PhD*

^1^Psychology Department, Université de Montréal, Montreal, QC; ^2^Center for Advanced Research in Sleep Medicine, Research center of the CIUSS du Nord-de-l’Ile-de-Montreal Montréal, QC; ^3^Department of Brain and Behavioral Sciences, University of Pavia, Pavia, Italy; ^4^Department of Neurology and Neurosurgery, McGill, Montréal, QC; ^5^McGill University Health Center, Montréal, QC; ^6^Department Psychiatry, Université de Montréal, Montréal, QC; ^7^Department of Electrical Engineering, École de Technologie Supérieure, Montréal, QC; ^8^Centre de Recherches Mathématiques, Université de Montréal, Montréal, QC

^$^Authors contributed equally.

^*^Corresponding Authors

**Corresponding Authors**

Julie Carrier, *PhD*

+1-514-343-6537

[julie.carrier.1@umontreal.ca](mailto:julie.carrier.1@umontreal.ca).

5400 Gouin West Blvd.

Center for Advanced Research in Sleep Medicine, Research Center—Hôpital du Sacré-Cœur de Montréal

Montréal, Québec, H4J 1C5, Canada.

Jean-Marc Lina *PhD*

+1-514-396-8688

[jean-marc.lina@etsmtl.ca](mailto:jean-marc.lina@etsmtl.ca)

Center for Advanced Research in Sleep Medicine, Research Center—Hôpital du Sacré-Cœur de Montréal

Montréal, Québec, H4J 1C5, Canada.

**ORCID**

| C. William Yao | 0000-0002-7234-7375 | Ronald B. Postuma | 0000-0002-6468-4734 |
| --- | --- | --- | --- |
| Giuseppe Fiamingo | 0000-0002-5030-6425 | Jacques Y. Montplaisir | 0000-0002-5585-9811 |
| Karine Lacourse | 0000-0001-8488-7312 | Jean-Marc Lina | 0000-0003-3600-601X |
| Sonia Frenette | 0009-0003-9486-129X | Julie Carrier | 0000-0001-5311-2370 |

**Short Title**: REM Sleep Microstructure Classification

**Keywords**: Rapid Eye Movement; REM Sleep Microstructures; Scoring; RBD

**Supplementary Materials**

***EDFbrowser and Display Setup***

Building on the goal of equity, the scoring techniques and guidance illustrated from our study was developed using EDFbrowser, an open-source application.(Beelen, 2008) Developed by Teunis van Beelen, EDFbrowser allows users to review, annotate and process polysomnography or relevant data files in the format of European data format (EDF) or a Glyph bitmap distribution format (BDF). To facilitate future users implementing the scoring techniques proposed, we have provided a brief step-by-step instruction below. Further details regarding to the use of EDFbrowser can be found on the host website: <https://www.teuniz.net/edfbrowser/>.

***- Step 1. Installation and System Requirement***


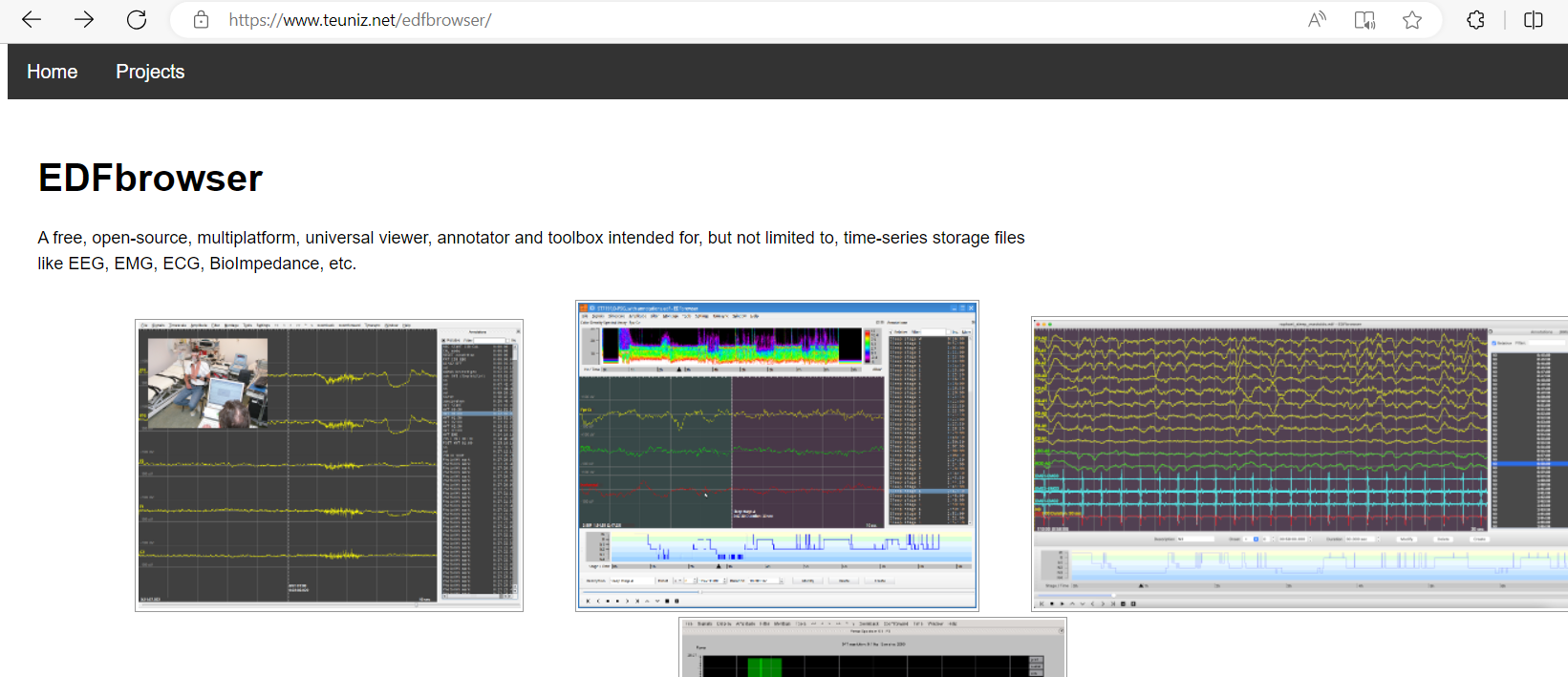


EDFbrowser supports easy installation for Microsoft Windows (2000, XP and later versions) and Linux. For Mac users, some additional compilation will be required.

***- Step 2. Standard Nomenclature***

To use the setting files provided, electrodes should be labeled using only the standard 10-20 system nomenclature in the recording file, as described in the official EDF website <https://www.edfplus.info/specs/edftexts.html>. Because the implementation is text-sensitive, any affix in the electrode labels should be removed unless it is included in the standard nomenclature. If the electrodes were already labeled in the standard nomenclature, please, proceed to Step 3. If not, we have provided step-by-step instructions below using Notepad++, an open-source text editor.

*- 2.1 Download and install Notepad++ from the official website:* [*https://notepad-plus-plus.org/*](https://notepad-plus-plus.org/)*.*

*
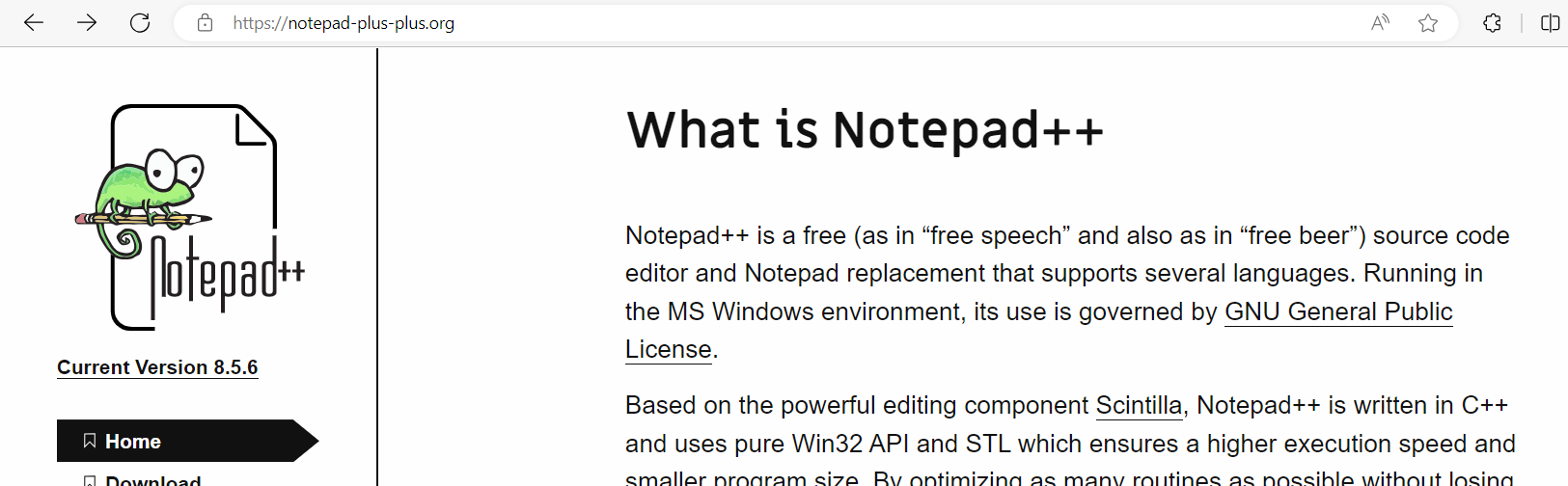
*

*- 2.2 Install HEX-Editor from the Plugin Admin tab in Notepad++*

*
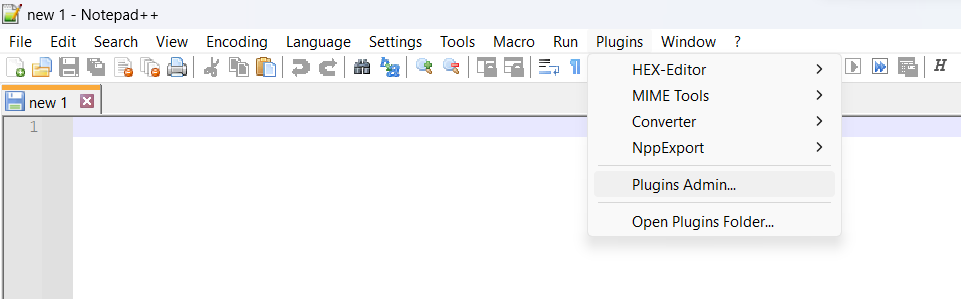
*

*
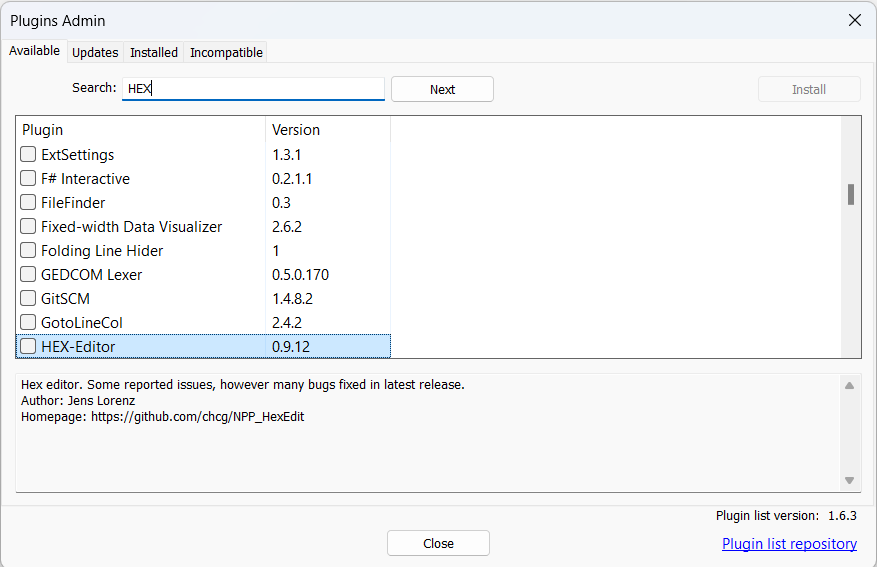
*

*-2.3 Open the EDF files in the HEX editor*

If the user has successfully opened the recording in the HEX editor, the recording should be displayed in similar format as the screenshot below.

*
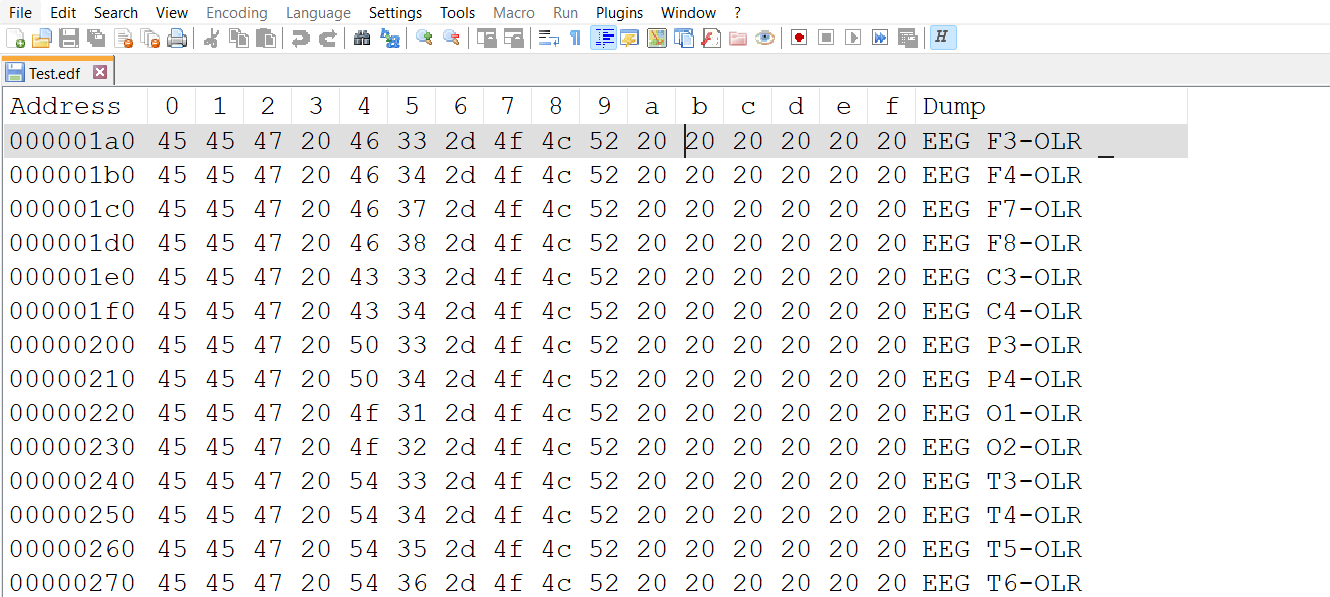
*

*-2.4 Save the File after Replacing the Affix with Empty Spaces*

Due to the structural design of the hexadecimal filing system, users will have to replace the affix (in hex nomenclature) with empty spaces, which equals to the same length as the affix (e.g., -OLR in the screenshot below). The suffix “-OLR” is coded in four paired indices: 2d 4f 4c 52, separated by space. To remove this suffix, we typed the corresponding codes in the search box and four “20” (i.e., empty space) in the box below. It is worth noting that since some affixes may be part of a word used in documentation, the “Replace All” button should only be used if the “In selection” box is checked.

*
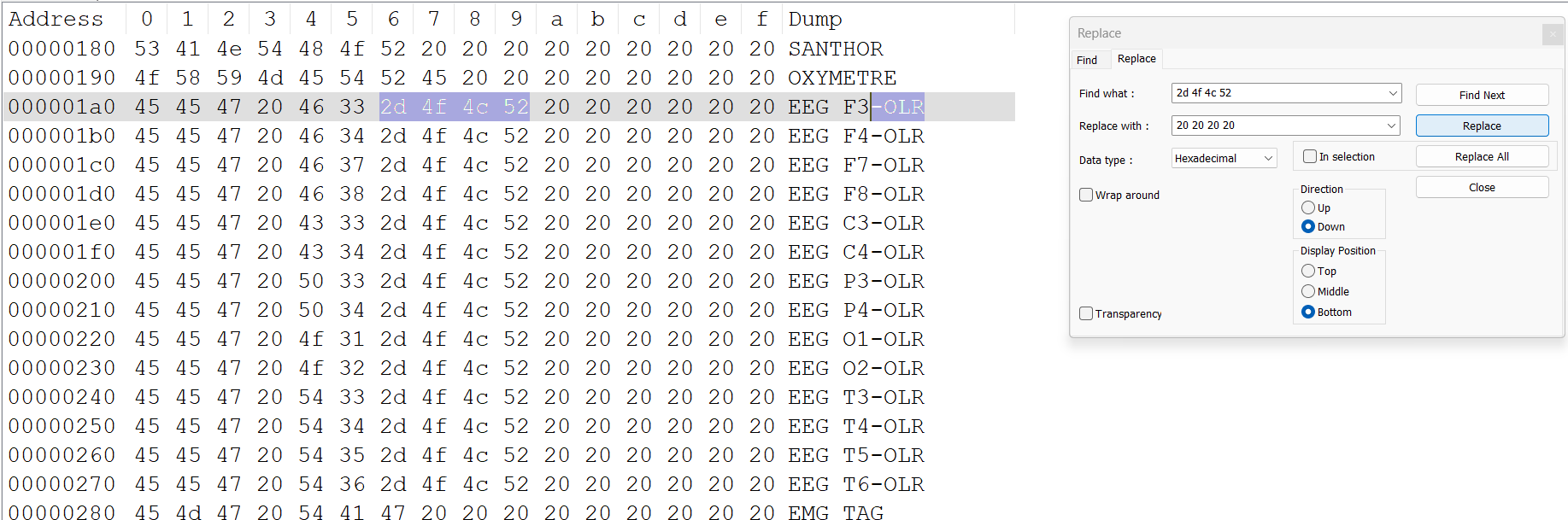
*

***- Step 3. Import Recordings***

***- Step 4. Download and Unzip Setting Files from the Journal Webpage***

Three setting files were included in the file. All three settings predefine a 30-second epoch as the display window length. All signals will be displayed at 100µV per centimetre. All channels of the standard 10-20 system were included in the naïve display setting (i.e., Naïve_30s_Epoch), The remaining two files containing EOG as a substring in the file names contained settings of filters and duplicated EOG channels as described in the manuscript. Further details about the *.mtg* file structure can be found on the EDFbrowser website:

<https://www.teuniz.net/edfbrowser/montage_file_format.txt>.

| ***File Name (.mtg)*** | ***Electrodes Included*** | | ***Amplitude Sensitivity*** | ***Filters*** |
| --- | --- | --- | --- | --- |
| Naïve_30s_Epoch | EOG | E1/2 | 100µV/cm | x |
|  | EEG | Fp1/2, Fz, F3/4, F7/8, Cz, C3/4, T3/4, Pz, P3/4, T5/6, O1/2 |  |  |
|  | Others | Cannula  SM, LAT, RAT  DI/II |  |  |
| EOG_Duplicate | EOG | E1/2 | 100µV/cm | x |
|  | EEG | F3/4, F7/8, C3/4, T3/4, O1/2 |  |  |
| Filtered_EOG_Duplicate |  |  | 100µV/cm | Serial Pass-Filter (1-5Hz) |
|  | Others | chin, RAT |  |  |

*Abbreviation:*

*SM: submentalis*

*LAT: left anterior tibialis*

*RAT: right anterior tibialis*

*DI/II: lead 1 or 2 ECG*

***- Step 5. Assign Fast Key Switch for the Settings***

Navigate the mouse cursor to the *Montage* tab on the top task bar. Click on the option called “Edit key-bindings for montages”. Next, navigate to the folder where the setting files were stored to link the function keys to the corresponding setting files. After the window is closed, users will be able to switch between the settings via the function keys as assigned.


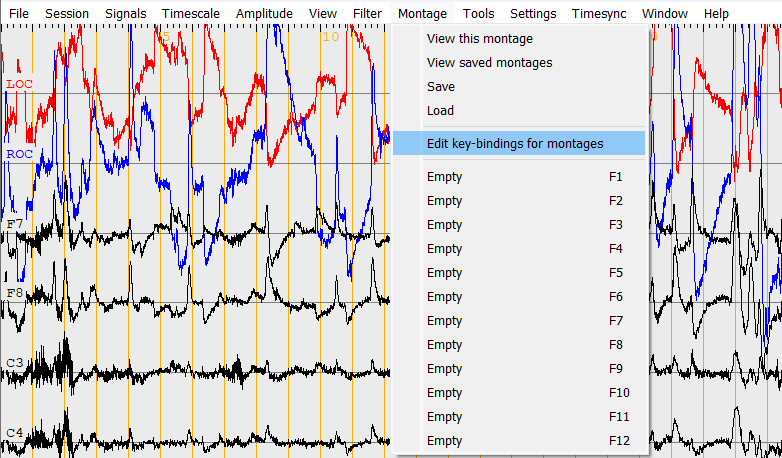

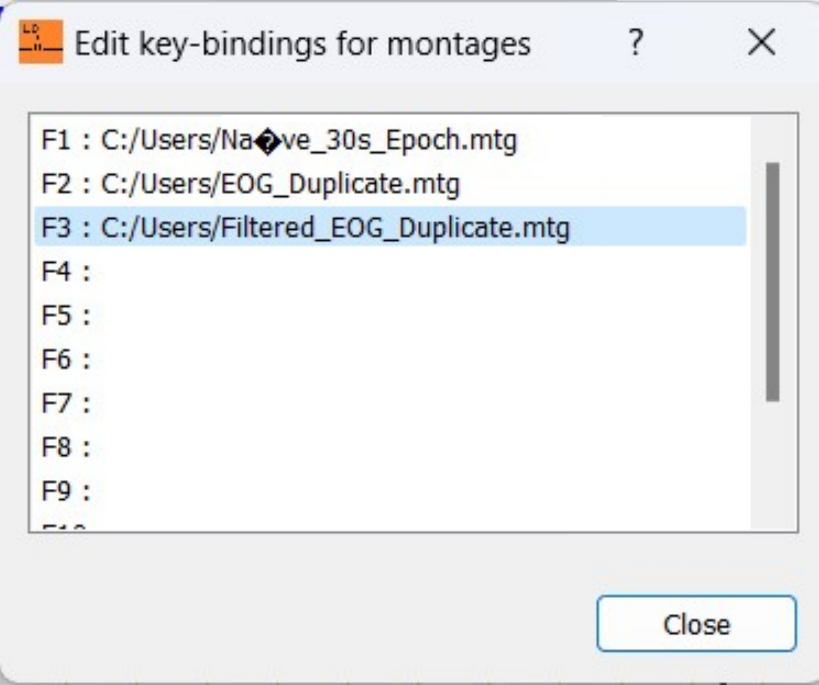


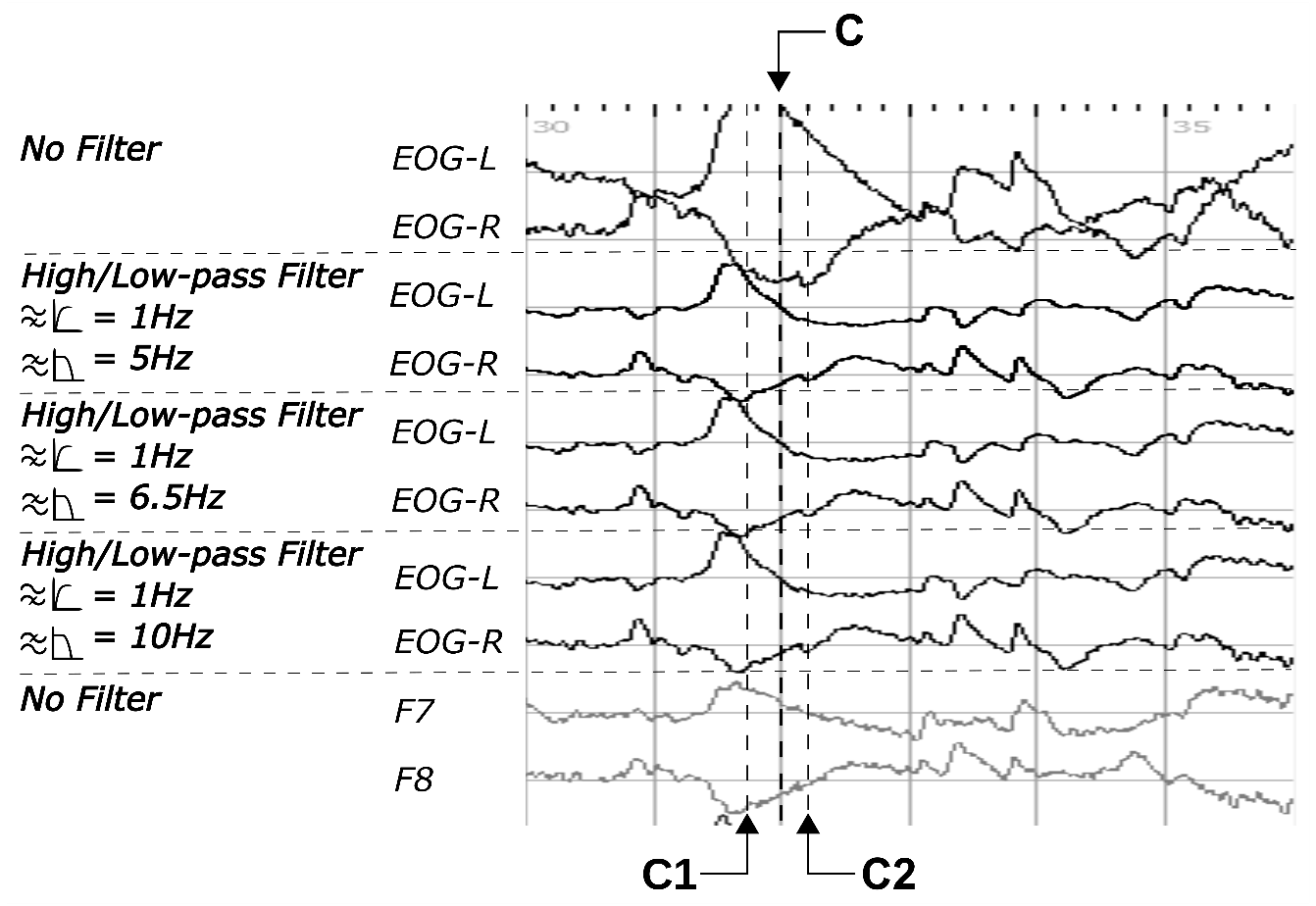


**Figure s1.** An example of a pitfall in the use of filters

The figure above showed a zoom-in image of Figure 6. The deflection of REM from left to right was annotated for **C1**) the roll-off point of the initial deflection, **C**) the maximum amplitude of the deflection, and **C2**) the second peak on the right EOG channel. Temporally, the filter-induced phase shift would cause a delay in the occurrence of a wave (i.e., right shift) whereas the deflection above appeared to be left-shifted. When examined closely, one may notice the major change in shape occurred between C1 and C, where a slowing in REM was noted. Due to the effect of the high-pass filtering component, the slowing in the deflection was suppressed and left with a pronounced deceleration.

**
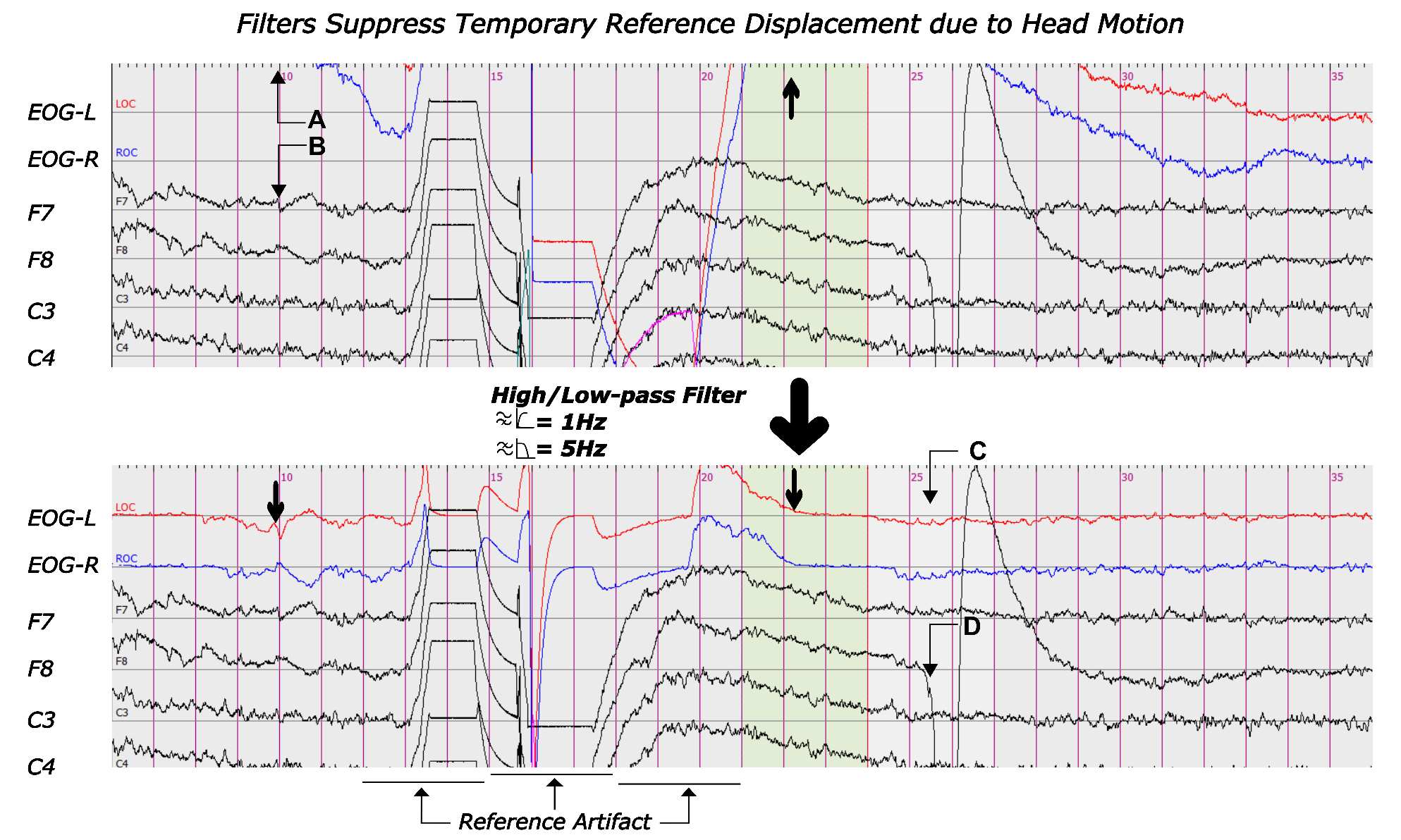
**

**Figure s2.** An example of the use of the serial high/low-pass filter

A temporary reference displacement occurred after a head turn due to dream enactment behavior. The serial high/low-pass filter was able to facilitate REM detection without suppressing the signals by increasing amplitude sensitivity and saved time from scrolling vertically on the display window. A) eye movements missing from the display window due to artifacts B) a proxy of eye movement at the frontal leads C) corrected EOG signals after applying filters D) unusable proxy due to artifacts.

**
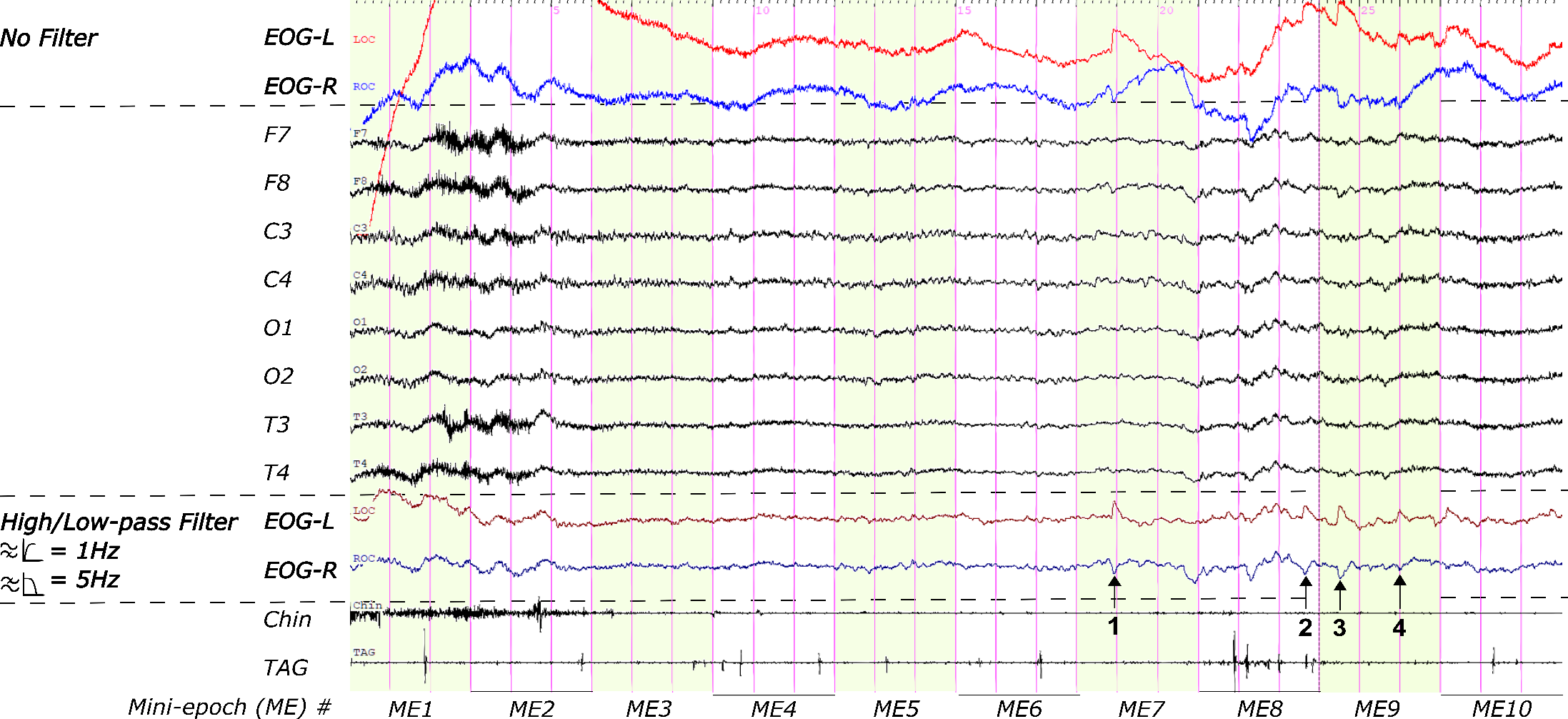
**

**Figure s3.** An example of the use of the proposed solutions

The figure displayed the use of the proposed solutions and the setting files using the example tracings illustrated at the lower panel of Figure 2. The first two tracings in red and blue illustrated the original EOG tracings with clear interferences of multiple artifacts. The same pair in the later segment were filtered through the serial high-low-pass filter as one of the proposed solutions. Filtered signals revealed four REM signals that were otherwise masked by the artifacts in the original tracings.

**
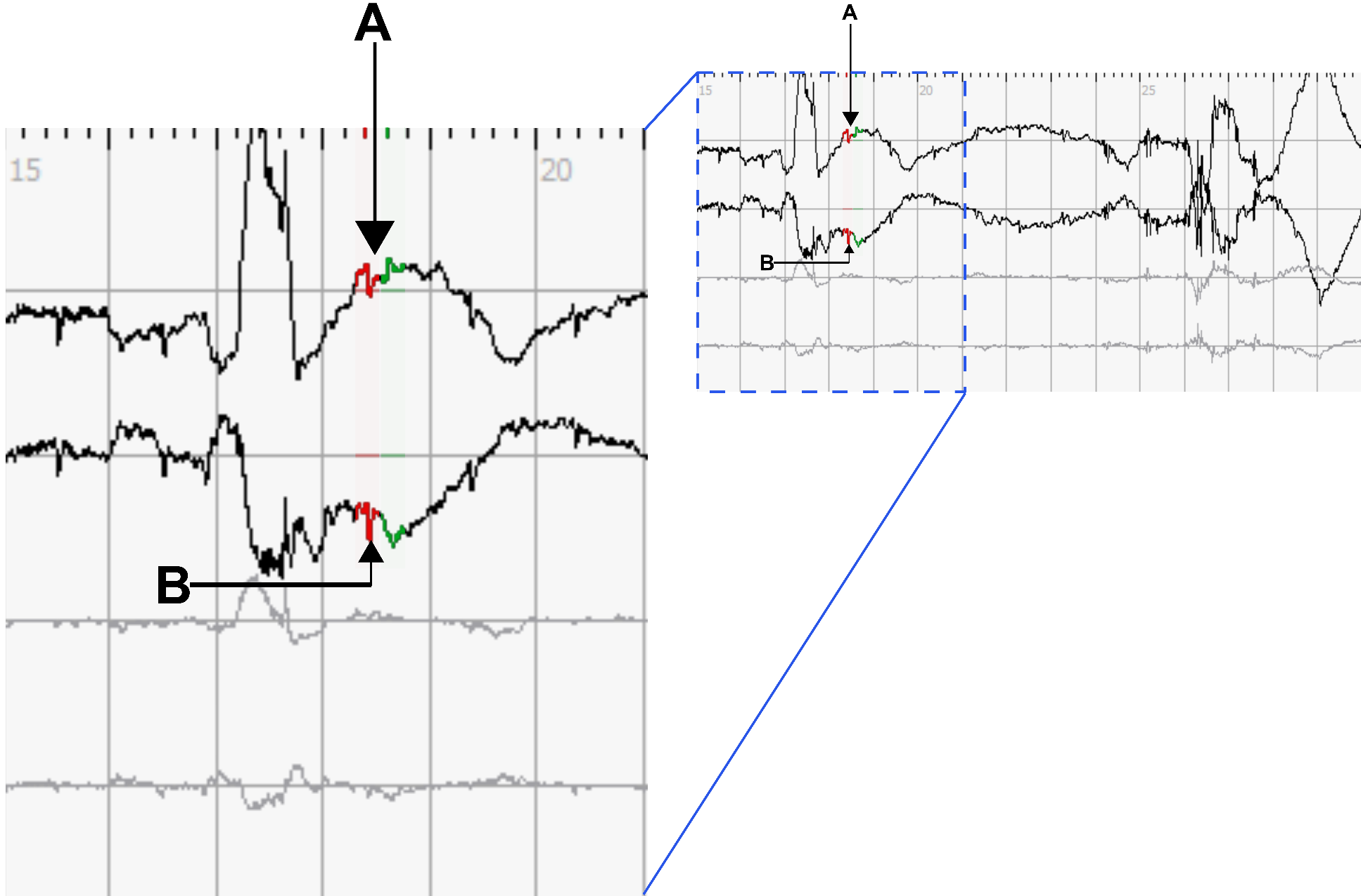
**

**Figure s4.** EKG artifact alters the shape of an eye movement.

The graph above illustrated the potential mild influence of a downward QRS complex as an EKG artifact in **A**) the left and **B**) the right EOG channels. On the left EOG channel, the downward R wave appeared to be `pressing` into an ongoing deflection. Followingly, the S wave could be seen distorting an ongoing deflection in the left EOG channel whereas distortion was minimal in the right EOG channel.

**e-References**

Beelen, T. v. (2008). *EDFbrowser*. Teunis van Beelen. <https://www.teuniz.net/edfbrowser/?trk=public_profile_project-button>
